# Design, synthesis, and exploration of antibacterial activity of 6*H*-1,2-oxazin-6-ones †

**DOI:** 10.1039/d4ra04220d

**Published:** 2024-07-29

**Authors:** Eleazar Alcántar-Zavala, Francisco Delgado-Vargas, Fabricio Marín-González, Gabriela López Angulo, Hugo Enrique Aguirre-Madrigal, Adrián Ochoa-Terán, Gibrán Rodríguez-Vega, Gerardo Aguirre-Hernández, Julio Montes-Avila

**Affiliations:** a Facultad de Ciencias Químico-Biológicas, Universidad Autónoma de Sinaloa Culiacán 80010 Sinaloa Mexico; b Programa de Posgrado en Ciencias Biomédicas, Facultad de Ciencias Químico-Biológicas, Universidad Autónoma de Sinaloa Culiacán 80010 Sinaloa Mexico jmontes@uas.edu.mx; c Centro de Graduados e Investigación en Química, Tecnológico Nacional de México/Instituto Tecnológico de Tijuana Tijuana 22444 Baja California Mexico; d Unidad Académica de Ciencias Químico-Biológicas y Farmacéuticas, Universidad Autonóma de Nayarit Tepic 63155 Nayarit Mexico

## Abstract

This study reports the *in silico* design of 30 6*H*-1,2-oxazin-6-ones against DHFR and PTC antimicrobial targets. Docking compounds 1, 3, 4, 6, and 8 with both enzymes was favorable, outperforming Trimethoprim with DHFR. Therefore, 12 6*H*-1,2-oxazin-6-ones, including the most promising compounds, were synthesized through an aminolysis reaction of β-cyanoketones with hydroxylamine hydrochloride, obtaining moderate to high yields (55–88%). Subsequently, antibacterial studies were conducted against five bacteria: four Gram-positive MRSA (ATCC 43300 and three clinical isolates) and one Gram-negative (*E. coli* ATCC 25922). Compounds 1, 2, 3, 4, 6, and 8 inhibited bacterial growth with MIC values ranging from 3.125 to 200 μg mL^−1^. Compound 1 showed better activity against Gram-positive bacteria than Linezolid. Toxicity assays indicated no adverse effects of the active oxazinones *in silico* and *in vitro*. This study demonstrated the antibacterial potential of the selected 6*H*-1,2-oxazin-6-ones against resistant human pathogenic bacteria.

## Abstract 2

This study reports the *in silico* design of 30 6*H*-1,2-oxazin-6-ones against DHFR and PTC antimicrobial targets.

## Graphical abstract

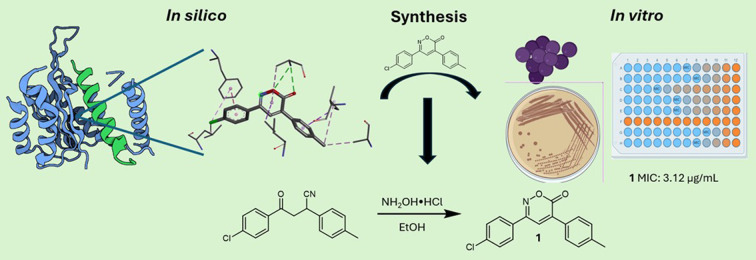

## Introduction

Currently, computational technologies largely support the design and search for new biologically active compounds. These technologies provide data generation and evaluation tools, predicting the interaction between two molecules through a drug-receptor binding model. This approach makes syntheses more directed, reducing costs and experimental time.^1,2^

6*H*-1,2-Oxazin-6-ones are heterocyclic compounds traditionally synthesized through the condensation of oximes with 4-oxocarboxylic acids and their derivatives under general reaction conditions, such as protic polar solvents, basic media, and long reaction times.^3–6^ Grignard reagents, metal catalysts, phosphorus compounds, and reactions involving nitro compounds have been useful options in synthesizing these heterocycles.^7–20^

While these processes offer different synthesis variants, most have disadvantages, such as numerous reaction steps, expensive reagents, the use of metallic catalysts and bases, long reaction times, and yields ranging from low to moderate.^3,7–20^

6*H*-1,2-Oxazin-6-ones showed a wide range of biological properties, including modulation of glucocorticoid receptors, anticancer, antifungal, and antibacterial activities.^3,21–24^ However, their high antibacterial capacity is supported by a relatively limited number of studies.^4–6,25^ Within this context, infections caused by bacteria resistant to conventional antibiotics are a leading cause of death worldwide and are projected to become the leading cause in the coming years, surpassing chronic-degenerative diseases such as cancer and diabetes. Thus, new therapeutic alternatives must be developed to address this serious public health problem.^26–30^

Given the lack of previous information on oxazinones docking against any therapeutic target and considering their structural similarities to the antibiotic Linezolid, it was decided to perform docking studies with the ribosomal phosphoryl transferase center (PTC) located in the 50s ribosomal subunit, which is involved in bacterial protein synthesis.^26,31^ Additionally, considering the precursor's affinity for the enzyme dihydrofolate reductase (DHFR), docking studies with DHFR were also of interest. DHFR is a cofactor involved in the biosynthesis of purines and thymidine by maintaining the metabolic levels of tetrahydrofolate (THF).^32,33^

Therefore, developing better synthetic routes to obtain the core of 6*H*-1,2-oxazin-6-ones as precursors of high-potency antibacterials is important. In this regard, β-cyanoketones are key intermediates in the synthesis of various heterocycles^34^ due to their ketone group, which can be converted to oxime and nitrile groups; these groups are versatile and can be transformed into different classes of biologically active molecules.^35–38^ Our research group has prepared β-cyanoketones from chalcones, providing important precursors for producing 6*H*-1,2-oxazin-6-ones.^32^

This study began with the *in silico* design of 6*H*-1,2-oxazin-6-ones and the evaluation of their potential interactions with the therapeutic targets DHFR and PTC. The best candidates were then synthesized from the reaction of β-cyanoketones with hydroxylamine hydrochloride in the presence of a protic solvent such as ethanol, without the use of bases or additives traditionally employed. The synthesized compounds were then subjected to *in vitro* studies to explore their potential as antibacterial agents against clinically important resistant bacteria.

## Experimental

### In silico

The three-dimensional structures of the two receptors (dihydrofolate reductase [DHFR] from *Mycobacterium tuberculosis* and ribosomal RNA from *E. coli*) were retrieved from the Protein Data Bank (https://www.rcsb.orq/pdb/home/home.do) using the PDB IDs 1DG5 and 4V4Q. The receptors were prepared for molecular docking using Chimera software,^39^ where the water molecules, ions, and other co-crystallized ligands were removed using the protein preparation module. The 12 compounds were analyzed using the SwissADME server for their predicted pharmacokinetic parameters: absorption, distribution, metabolism, and excretion (ADME).^40^ Subsequently, non-polar hydrogens were eliminated, and Gasteiger–Marsili charges were added through the AutoDock Vina^41,42^ program and its graphical interface AutoDockTooIs 1.54 (ADT). The ADT program and the AutoGrid 4.2 auxiliary program were used to generate the search box or grid, positioning in place the co-crystallized ligand of the DHFR enzyme. Considering the ribosomal RNA of *E. coli*, the binding site for PTC was located. The conformations accessible by the ligands were established using AutoDock Vina with an exhaustiveness of 32 runs. Finally, the best relaxed ligand–enzyme docking model was selected based on free-energy minimization, determined by analyzing the binding interactions of the selected best-docked poses. Two-dimensional docking maps were created and designed using the PoseView server^43,44^ and the Discovery Studio Visualizer software.^45^ The molecular docking protocol was validated by re-docking the co-crystallized ligand of the DHFR enzyme and evaluating the RMSD value of its highest-ranking docked pose. The docking resulted in an RMSD value of 0.937 Å.

### Molecular dynamic studies

Ligand–protein complexes that displayed generally better docking poses were subjected to molecular dynamic (MD) simulation. The study was performed using GROMACS 2023.3 (ref. 46) version. Compounds 1, 4, 6, and 8 were subjected to 100 ns dynamic simulations in an explicit water model using CHARMM27 (ref. 47) force field. The TIP3P model was used to represent water molecules.^48^ The topology file of the compounds was created *via* the SwissParam server.^49^ To mimic the physiological conditions, 0.15 M salt (NaCl) was added. Before the production run, the protein system was equilibrated by running 100 ps of NVT (isothermal–isochoric) and NPT (isothermal–isobaric) ensemble. The simulation system was closely monitored to reach a temperature of 300 K and around one atmospheric pressure. Molecular dynamic simulations were then carried out in the triclinic box with a minimum distance of 1.0 nm between any protein atom and the walls of the box. Root mean square deviation (RMSD) of protein (backbone), root mean square fluctuation (RMSF) of amino acid residues, radius of gyration (rg), and hydrogen bonds were plotted using XMGRACE v5.1.19.^50^

### Toxicity prediction

The toxicity of the synthesized 6*H*-1,2-oxazin-6-ones was analyzed with the ProTox-II webserver,^51,52^ generating the following predicted parameters: median lethal dose (LD_50_) in rodents, organ toxicity (hepatotoxicity), as well as toxicity endpoints, including carcinogenicity, immunotoxicity, mutagenicity, and cytotoxicity.

### Chemistry

Reagents and solvents obtained from commercial suppliers were used without previous purification. The nuclear magnetic resonance was carried out in a Bruker Advance III HD 400 MHz equipped with an internal reference of tetramethylsilane (TMS). The FTIR spectra were recorded on a PerkinElmer Spectrum 400 FTIR spectrometer. The melting points were obtained in an electrothermal apparatus. The electron ionization mass spectra (EIMS) were obtained on the Agilent Technologies 5975 GC/MS system.

### Synthesis of 6*H*-1,2-oxazin-6-ones

The β-cyanoketones (1.0 mmol), 8 mmol hydroxylamine hydrochloride, and 20 mL ethanol were mixed and heated at reflux temperature for 24 h. The product was extracted with ethyl acetate, dried over anhydrous Na_2_SO_4_, and concentrated under reduced pressure. Finally, the 6*H*-1,2-oxazin-6-one was purified by column chromatography on silica gel using hexane–ethyl acetate (9 : 1 v/v) as eluent.

### 3-(4-Chlorophenyl)-5-(*p*-tolyl)-6*H*-1,2-oxazin-6-one (1)

Orange solid, 55% yield (163.7 mg, 0.5 mmol). M.p. 114–116 °C. FTIR: 3059, 2922, 1702, 1597, 1494, 815 cm^−1^. ^1^H NMR (400 MHz, CDCl_3_): *δ* 7.75 (d, *J* = 8.5 Hz, 2H), 7.73 (d, *J* = 8.1 Hz, 2H), 7.48 (d, *J* = 8.5 Hz, 2H), 7.45 (s, 1H), 7.29 (d, *J* = 8.0 Hz, 2H), 2.41 (s, 3H). ^13^C NMR (101 MHz, CDCl_3_): *δ* 163.23, 153.74, 141.62, 137.43, 136.06, 130.41, 129.59, 129.51, 128.81, 128.68, 127.92, 124.97, 21.45. MS(IE) *m*/*z*: 297 (90), 239 (100), 202 (20).

### 3-Phenyl-5-(*p*-tolyl)-6*H*-1,2-oxazin-6-one (2)

Orange solid, 60% yield (144.8 mg, 0.55 mmol) M.p. 130–132 °C. FTIR: 3038, 2918, 1724, 1604, 1494, 1115 cm^−1^. ^1^H NMR (400 MHz, DMSO-*d*_6_): *δ* 7.99 (m, 3H), 7.88 (d, *J* = 8.2 Hz, 2H), 7.58 (m, 3H), 7.34 (d, *J* = 8.0 Hz, 2H), 2.4 (s, 3H). ^13^C NMR (100 MHz, DMSO-*d*_6_): *δ* 163.6, 155.2, 140.9, 134.9, 132.1, 131.4, 129.5, 129.49, 129.41, 127.4, 127.1, 21.4. MS(IE) *m*/*z*: 263 (70), 205 (100), 116 (50).

### 3,5-Bis(4-chlorophenyl)-6*H*-1,2-oxazin-6-one (3)

White solid, 55% yield (167.2 mg, 0.52 mmol). M.p. 202–204 °C. FTIR: 3063, 1700, 1691, 1593, 1492, 1095, 826 cm^−1^. ^1^H NMR (400 MHz, DMSO-*d*_6_): *δ* 8.10 (s, 1H), 8.03 (d, *J* = 8.6 Hz, 2H), 7.99 (d, *J* = 8.6 Hz, 2H), 7.64 (d, *J* = 8.6 Hz, 2H), 7.61 (d, *J* = 8.6 Hz, 2H). ^13^C NMR (100 MHz, DMSO-*d*_6_): *δ* 163.2, 154.3, 136.5, 135.9, 133.9, 131.5, 131.0, 130.8, 129.6, 129.3, 128.9, 128.0. MS(IE) *m*/*z*: 318 (60), 282 (30), 258 (100), 189 (15), 135 (30).

### 5-(4-Chlorophenyl)-3-(*p*-tolyl)-6*H*-1,2-oxazin-6-one (4)

White solid, 58% yield (164.5 mg, 0.55 mmol). M.p. 166–168 °C. FTIR: 3063, 2921 1723, 1611, 1491, 1092, 816 cm^−1^. ^1^H NMR (400 MHz, DMSO-*d*_6_): *δ* 8.06 (s, 1H), 7.98 (d, *J* = 8.7 Hz, 2H), 7.89 (d, *J* = 8.2 Hz, 2H), 7.60 (d, *J* = 8.7 Hz, 2H), 7.38 (d, *J* = 8.1 Hz, 2H), 2.40 (s, 3H). ^13^C NMR (100 MHz, DMSO-*d*_6_): *δ* 163.4, 154.9, 141.5, 135.8, 133.9, 131.4, 131.1, 130.1, 129.0, 128.9, 128.2, 21.4. MS(IE) *m*/*z*: 297 (80), 262 (40), 239 (100), 136 (45).

### 5-(4-Chlorophenyl)-3-phenyl-6*H*-1,2-oxazin-6-one (5)

Pink solid, 55% yield (148.3 mg, 0.52 mmol). M.p. 142–144 °C. FTIR: 3057, 1723, 1617, 1491, 1094, 830 cm^−1^. ^1^H NMR (400 MHz, DMSO-*d*_6_): *δ* 8.09 (s, 1H), 8.00 (m, 4H), 7.61 (m, 3H). ^13^C NMR (100 MHz, DMSO-*d*_6_): *δ* 163.4, 155.2, 135.9, 133.9, 131.9, 131.5, 131.4, 129.5, 128.9, 128.3, 128.3, 127.5. MS(IE) *m*/*z*: 283 (60), 248 (40), 225 (100), 189 (35) 136 (40).

### 3,5-Di-*p*-tolyl-6*H*-1,2-oxazin-6-one (6)

Yellow solid, 88% yield (231 mg, 0.83 mmol). M.p. 164–166 °C. FTIR: 3057, 1723, 1617, 1491, 1094, 830 cm^−1^. ^1^H NMR (400 MHz, CDCl_3_): *δ* 7.76 (d, *J* = 7.9 Hz, 2H), 7.72 (d, *J* = 7.9 Hz, 2H), 7.51 (s, 1H), 7.33 (m, 4H). ^13^C NMR (101 MHz, CDCl_3_): *δ* 163.6, 154.5, 141.4, 141.3, 135.7, 129.9, 129.5, 129.09, 129.06, 128.6, 126.5, 125.6, 21.44, 21.42. MS(IE) *m*/*z*: 277 (60), 248 (40), 225 (100), 189 (35) 136 (40).

### 3-(4-Methoxyphenyl)-5-(*p*-tolyl)-6*H*-1,2-oxazin-6-one (7)

Green solid, 60% yield (167.5 mg, 0.57 mmol). M.p. 136–138 °C. FTIR: 3070, 2918, 1712, 1601, 1509, 1252, 1220 cm^−1^. ^1^H NMR (400 MHz, CDCl_3_): *δ* 7.74 (t, *J* = 8.1 Hz, 4H), 7.48 (s, 1H), 7.29 (d, *J* = 8.0 Hz, 2H), 7.26 (s, 1H), 7.02 (d, *J* = 8.9 Hz, 2H), 3.88 (s, 3H), 2.42 (s, 3H). ^13^C NMR (101 MHz, CDCl_3_): *δ* 163.61, 161.94, 154.08, 141.29, 135.68, 129.51, 129.10, 128.67, 128.13, 125.62, 124.18, 114.65, 55.47, 21.44. MS(IE) *m*/*z*: 293.1 (67), 235.1 (100), 192.1 (33), 116.0 (52.8).

### 5-(4-Methoxyphenyl)-3-(*p*-tolyl)-6*H*-1,2-oxazin-6-one (8)

Yellow solid, 74% yield (206.7 mg, 0.70 mmol). M.p. 132–134 °C. FTIR: 3062, 2922, 1713, 1602, 1508, 1247, 1122 cm^−1^. ^1^H NMR (400 MHz, CDCl_3_): *δ* 7.84 (d, *J* = 8.9 Hz, 2H), 7.68 (d, *J* = 8.2 Hz, 2H), 7.44 (s, 1H), 7.30 (d, *J* = 8.0 Hz, 2H), 6.98 (d, *J* = 8.9 Hz, 2H), 3.86 (s, 3H), 2.42 (s, 3H). ^13^C NMR (101 MHz, CDCl_3_): *δ* 163.77, 161.83, 154.56, 141.39, 135.10, 130.42, 129.91, 129.20, 126.52, 124.53, 124.17, 114.29, 55.46, 21.41. MS(IE) *m*/*z*: 293.1 (100), 235.1 (82), 132.1 (73).

### 5-(4-Chlorophenyl)-3-(4-methoxyphenyl)-6*H*-1,2-oxazin-6-one (9)

Yellow solid, 65% yield (184.4 mg, 0.58 mmol). M.p. 160–162 °C. FTIR: 3074, 2923, 1700, 1606, 1519, 1242, 1093, 827 cm^−1^.^1^H NMR (400 MHz, DMSO-*d*_6_): *δ* 8.06 (s, 1H), 7.97 (d, *J* = 8.7 Hz, 2H), 7.95 (d, *J* = 9.0 Hz, 2H), 7.60 (d, *J* = 8.7 Hz, 2H), 7.11 (d, *J* = 9.0 Hz, 2H), 3.85 (s, 3H). ^13^C NMR (100 MHz, DMSO-*d*_6_): *δ* 163.4, 162.0, 154.5, 135.7, 133.8, 131.5, 131.2, 129.0, 128.9, 128.3, 124.0, 114.9, 55.6. MS(IE) *m*/*z*: 313 (70), 255 (100), 225 (100), 212 (15), 176 (17), 133 (20).

### 3-(4-Chlorophenyl)-5-(4-methoxyphenyl)-6*H*-1,2-oxazin-6-one (10)

Yellow solid, 61% yield (173.0 mg, 0.55 mmol). M.p. 174–176 °C. FTIR: 3074, 2923, 1711, 1606, 1511, 1242, 1093, 827 cm^−1^.^1^H NMR (400 MHz, DMSO-*d*_6_): *δ* 7.81 (d, *J* = 8.6 Hz, 2H), 7.67 (d, *J* = 8.7 Hz, 2H), 7.53 (d, *J* = 8.5 Hz, 2H), 7.09 (s, 1H), 7.05 (d, *J* = 8.7 Hz, 2H), 3.80 (s, 3H). ^13^C NMR (101 MHz, DMSO-*d*_6_): *δ* 163.64, 159.42, 155.71, 136.36, 135.33, 131.34, 129.48, 127.99, 126.99, 125.43, 114.90, 106.77, 55.70. MS(IE) *m*/*z*: 313.1 (55), 255 (45), 176.1 (20), 132.1 (70), 40 (100).

### 5-(3-Methoxyphenyl)-3-(*p*-tolyl)-6*H*-1,2-oxazin-6-one (11)

Gray solid, 70% yield (198.6 mg, 0.67 mmol). M.p. 88–90 °C. FTIR: 3040, 2987, 1717, 1571, 1416, 1259, 119 cm^−1^. ^1^H NMR (400 MHz, DMSO-*d*_6_): *δ* 7.68 (d, *J* = 8.2 Hz, 2H), 7.50 (s, 1H), 7.41–7.34 (m, 3H), 7.30 (d, *J* = 8.0 Hz, 2H), 7.07–6.99 (m, 1H), 3.85 (s, 3H), 2.42 (s, 3H). ^13^C NMR (100 MHz, DMSO-*d*_6_): *δ* 163.37, 159.72, 154.48, 141.56, 135.71, 133.19, 129.96, 129.85, 128.94, 126.61, 126.52, 121.03, 116.49, 114.41, 55.46, 21.42. MS(IE) *m*/*z*: 293.2 (65), 235.2 (55), 192.1 (32), 132.1 (100), 119.1 (30), 89.1 (25).

### 3-(3-Methoxyphenyl)-5-(*p*-tolyl)-6*H*-1,2-oxazin-6-one (12)

Purple solid, 57% yield (161.7 mg, 0.55 mmol). M.p. 92–94 °C. FTIR: 3068, 2923, 1729,1606, 1447, 1266, 1119 cm^−1^. ^1^H NMR (400 MHz, DMSO-*d*_6_): *δ* 8.01 (s, 1H), 7.88 (d, *J* = 8.2 Hz, 2H), 7.52–7.47 (m, 2H), 7.43 (t, *J* = 7.9 Hz, 1H), 7.37 (d, *J* = 8.0 Hz, 2H), 7.16–7.06 (m, 1H), 3.83 (s, 3H), 2.39 (s, 3H). ^13^C NMR (101 MHz, DMSO-*d*_6_): *δ* 163.46, 159.58, 154.96, 141.49, 134.87, 133.59, 130.10, 129.93, 129.16, 128.16, 127.34, 121.92, 116.49, 115.18, 55.81, 21.41. MS(IE) *m*/*z*: 293.2 (70), 235.2 (55), 192.1 (32), 132.1 (100), 89.1 (25).

### Antibacterial activity of 6*H*-1,2-oxazin-6-ones

The antibacterial activity of all compounds was evaluated against five bacterial strains, four Gram-positive MRSA (ATCC 43300 and three clinical isolates) and one Gram-negative (*E. coli* 25 922). All bacteria were cultured in Muller–Hinton broth. The Minimal Inhibitory Concentration (MIC) was determined by the broth microdilution method according to the recommendations of the National Committee for Clinical Laboratory Standards. The bacteria were cultured for 18–20 h to obtain about 10^8^ CFU mL^−1^ of bacteria. The culture was diluted to 10^6^ CFU mL^−1^ in Muller–Hinton broth for the antibacterial assay in 96-well ELISA-type dishes. The compounds were first evaluated at 200 μg mL^−1^ against Gram-positive and Gram-negative strains. Then, the active compounds were evaluated at lower concentrations in the 200–1.17 μg mL^−1^ range.^26,53^

### Acute toxicity assay (*Artemia salina*)

The acute toxicity of the evaluated compounds was determined by inhibiting the mobility of the crustacean *A. salina* after 24 and 48 h of exposure, according to the procedure established by the OECD (organization for economic cooperation and development). The *A. salina* eggs were hatched in artificial seawater prepared with 38 g L^−1^ of sea salt (Instant Ocean®, Blacksburg, VA, USA) and oxygenated with an aquarium pump. The temperature was kept at 25–30 °C and under a light source (white neon, 70 W) for 48 h. The compounds were dissolved in Tween 80%, and four dilutions were prepared (1000, 500, 100, and 10 ppm). The toxicity assay was carried out in 4 mL test tubes, and each concentration was evaluated in triplicate. First, ten nauplii were added to each tube in a volume not exceeding 0.5 mL using a Pasteur pipette, then 100 μL of a compound dilution was added, and the final volume was adjusted to 2 mL with seawater. The tubes were then incubated at 25 °C for 24 h under artificial light, and the dead and live nauplii were counted; each tube was added 50 μL of formaldehyde (10% v/v) and let stand for 15 min to kill the remaining live nauplii. Finally, the total number of nauplii per tube was counted. The results were reported as mortality percentages, determined by the correction of the Abbott formula:^54,55^% Mortality = (DLT/ALT) × 100%where: DLT is the number of dead larvae in the tube, and ALT is the number of alive larvae in the tube.

## Results and discussion

The docking simulations were performed by optimizing the free energy binding of the compounds at the DHFR binding site of *Mycobacterium tuberculosis* (PDB 1DG5) and the PTC ribosomal RNA of *E. coli* (PDB 4V4Q). The compounds docked into the active site of these enzymes produced similar binding patterns and positioning at the binding site as antimicrobials Trimethoprim and Linezolid. The docking score (free binding energy, Δ*G*°, kcal mol^−1^) of the oxazinones with the enzymes is presented in Table 1. Docking was conducted for 30 different 6*H*-1,2-oxazin-6-ones, whose structures included various substitutions in the aromatic rings. Surprisingly, the best results were obtained for compounds with alkyl, methoxy, and chlorine groups (1–15 Table 1) over those presenting hydroxyl groups in their structure (16–30 Table S1†), which could act as acceptors of one or two hydrogen bonds through the heterocycle or did not generate them. In contrast, the high number of hydrophobic interactions was the driving force behind favorable couplings. These results agree with the literature, where active β-cyanoketones and oxazinones have aryl groups bearing halogen, methoxy, or alkyl substituents in their structure.^6,24,32,56^ The compounds demonstrated acceptable pharmacokinetic parameters (Table S2†) and passed Lipinski's rule-of-five test.^40^

**Table 1:** Docking scores of designed oxazinones with DHFR enzyme and the PTC

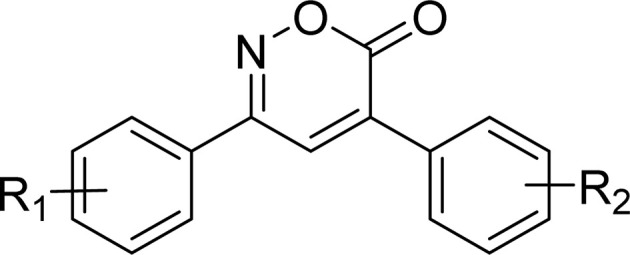				
			DHFR	PTC
6*H*-1,2-Oxazin-6-ones	R_1_	R_2_	Score (Δ*G*, kcal mol^−1^)	Score (Δ*G*, kcal mol^−1^)
1	4-Cl	4-Me	−9.0	−8.0
2	–H	4-Me	−8.8	−8.1
3	4-Cl	4-Cl	−8.9	−8.0
4	4-Me	4-Cl	−8.7	−8.5
5	–H	4-Cl	−8.4	−8.0
6	4-Me	4-Me	−9.4	−8.0
7	4-OMe	4-Me	−8.8	−8.5
8	4-Me	4-OMe	−9.2	−8.3
9	4-OMe	4-Cl	−8.7	−8.2
10	4-Cl	4-OMe	−9.0	−8.2
11	4-Me	3-OMe	−9.2	−8.4
12	3-OMe	4-Me	−8.8	−8.3
13	4-Me	3-Cl	−8.9	−8.6
14	4-Cl	3-Cl	−8.9	−8.7
15	3-OMe	4-Cl	−8.6	−8.2
Linezolid				−8.9
Trimethoprim			−6.7	

The docking between each of the 30 6*H*-1,2-oxazin-6-ones and the DHFR enzyme showed better scores than Trimethoprim (more negative), with compounds 1, 6, 8, and 11 standing out. The overlapping of the docked poses for these compounds and Trimethoprim at the binding site showed a similar binding pattern and orientation as the co-crystallized ligand (Fig. S1 and S2†).

Trimethoprim interacts with the DHFR binding primarily through hydrogen bonding with Asp27, Ile94, and Ile5 and through π–π or other hydrophobic interactions with Phe31. The interaction with Phe31 was observed in all oxazinones; compounds 1, 6, and 8 showed the highest probabilities of interacting with the DHFR, establishing from 9 to 11 interactions with different amino acid residues. It is proposed that the main interactions of the compounds occur through the oxygen and carbonyl of oxazinone, forming hydrogen bonds with Ala7 and other hydrophobic interactions with Phe31, Ile20, and Ile14.

**Fig. 1:**
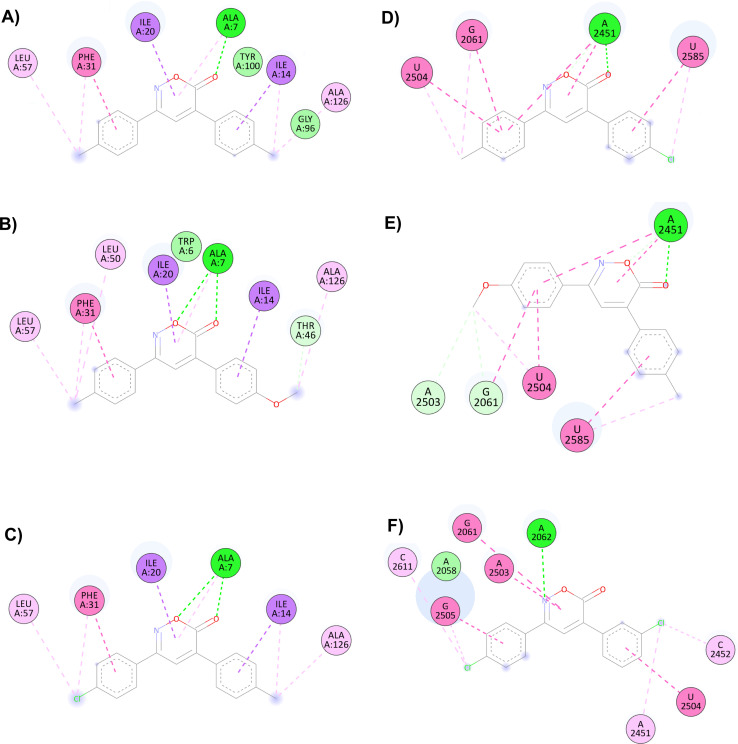
Interactions of 6*H*-1,2-oxazin-6-ones (A) 6, (B) 8 and (C) 1*versus* DHFR and (D) 4, (E) 7 and (F) 14 in the PTC.

The main interactions of docked compound 6 with DHFR include a hydrogen bond and a π–σ type interaction through the oxazinone and Ala7. Moreover, other hydrophobic interactions observed were π–π and π–σ type with Phe31, Ile14, and Ile20; as well as π–alkyl and alkyl interactions with Leu57, Phe31, Ile14, and Ala126 (Fig. 1A). Compound 8 forms two hydrogen bonds, a π–alkyl interaction of the heterocycle with Ala7, and π–π and π–σ interactions with Phe31, Ile20, and Ile14. Moreover, H–C, π–alkyl, and alkyl interactions with Thr46, Leu57, Phe31, Leu50, Ala7, and Ala126 are present (Fig. 1B). Similar to compound 8, compound 1 shows two hydrogen bonds and the π–alkyl interaction with Ala7, along with π–π and π–σ interactions with Phe31, Ile20, and Ile14. In contrast, the interaction with Thr46 and Leu50 was not observed due to the change in substituents (Fig. 1C).

For the docking of the 6*H*-1,2-oxazin-6-ones with the PTC binding site, a similar binding pattern, orientation, and overlap with that of Linezolid was observed (Fig. S3†). The most stable docked structures were obtained with 4, 7, 13, and 14, with score values similar to those calculated for Linezolid. Noteworthy, Linezolid's main interaction is a hydrogen bond between the acetamidomethyl group and the nucleotide G2505. Except for compound 7, this hydrogen bond interaction was not observed for the oxazinones in the study. However, G2505 generated hydrophobic interactions with some oxazinones, and interaction with A2451 was observed as a consistent pattern.

Compound 4 mainly interacts with A2451, showing two hydrogen bonds, one on the carbonyl and the other on the oxygen, along with two π–π interactions caused by the heterocycle and an aromatic ring. Additionally, π–π interactions of the aromatic rings with U2504, G2061, and U2585 are observed, with the substituents of the rings generating other π–alkyl hydrophobic interactions with the aforementioned nucleotides. Besides, the critical interaction between 4 and G2505 is not observed (Fig. 1D). Compound 7 presents three hydrogen bond interactions, the highest number in this study, involving the nucleotides G2505, A2451, and U2504. The carbonyl and the methoxy substituents mediate these interactions. Additionally, π–π and π–alkyl interactions are observed with A2451, C2452, U2585, and U2506 by the aromatic ring substituents (Fig. 1E). Compound 14 and PTC showed the most favorable docking score and distinctive interactions: a hydrogen bond generated by the heterocycle nitrogen with A2062, two π–π and π–alkyl interactions with G2505, a nucleotide recognized as the anchoring site of Linezolid, and a π–alkyl interaction with A2451, differing from the hydrogen bond interactions observed for other oxazinones. Additionally, compound 14 presented π–π and π–alkyl interactions with U2504, A2503, G2061, C2611, and C2452 (Fig. 1F).

### Chemistry

**Scheme 1:**
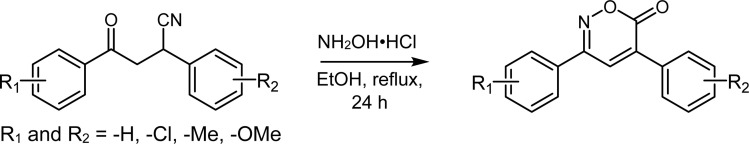
Synthetic route for the preparation of 6*H*-1,2-oxazin-6-ones from β-cyanoketones.

In this study, 12 out of the 30 designed 6*H*-1,2-oxazin-6-ones were successfully synthesized using a route starting with the aminolysis of β-cyanoketones with hydroxylamine hydrochloride (NH_2_OH·HCl), a reaction that targets the carbonyl (C=O) and nitrile (C≡N) functional groups of the β-cyanoketone (Scheme 1). The proposed mechanism for obtaining oxazinones involves two electron-deficient species at positions 1 and 4, such as the carbonyl and nitrile groups, and the generation of the oxime intermediate to form the heterocycle.^3^ The plausible synthetic process involves three reaction steps and is similar to what other authors have demonstrated.^3–5^ In the first step, the β-cyanoketone and hydroxylamine hydrochloride react in a protic polar solvent (EtOH : H_2_O) to form an oxime. In the second step, a tertiary carbanion is generated when the hydroxyl eliminates the asymmetric hydrogen, which resonates with the nitrile and is stabilized by forming a double bond. In the third step, the nitrile group is hydrolyzed, leading to the formation of a carboxylic acid or the formation of an amide, which further undergoes intramolecular esterification, resulting in the formation of the 6*H*-1,2-oxazin-6-one ring with the consequent loss of water or ammonium (Scheme S1†).

Finally, the 6*H*-1,2-oxazin-6-ones were purified *via* open-column chromatography, using silica gel as the stationary phase and a hexane/EtOAc (9 : 1) mixture as the mobile phase. The obtained yields were moderate to high, ranging from 55% to 88% (Table 2). The resulting products show at least one substitution (–Me, –OMe, –Cl) on the aromatic rings at positions 3 and 4. The oxazinones with higher yields contained electron-donating groups such as methoxy and methyl (6, 8, 11; 57–88%), while those with lower yields contained a chlorine atom, an electron-withdrawing group (1, 3, 4; 55–65%). These results are consistent with oxazinone yields lower than 70% reported in previous studies and the tendency for yields to be lower when halogen is the substituent.^18,32,57,58^ Only Gonçalves *et al.* (2016) and Shamala, D. and Shivashankar, K. (2017) reported yields greater than 90%, achieved using pyridine and DBU as catalysts under basic reaction conditions.^58,59^

**Table 2:** Results of obtaining 6*H*-1,2-oxazin-6-ones

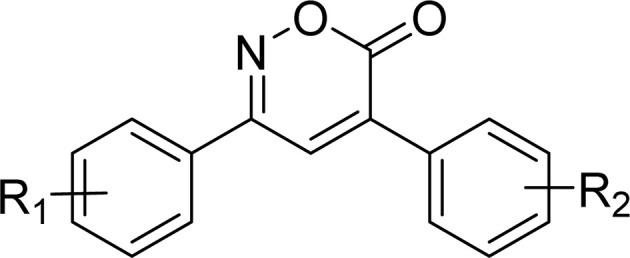			
6*H*-1,2-Oxazin-6-ones	R_1_	R_2_	Yield (%)
1	4-Cl	4-Me	55
2	–H	4-Me	60
3	4-Cl	4-Cl	55
4	4-Me	4-Cl	58
5	–H	4-Cl	55
6	4-Me	4-Me	88
7	4-OMe	4-Me	60
8	4-Me	4-OMe	74
9	4-OMe	4-Cl	65
10	4-Cl	4-OMe	61
11	4-Me	3-OMe	70
12	3-OMe	4-Me	57

The structure of the 12 6*H*-1,2-oxazin-6-ones was confirmed using FTIR, MS(EI), ^1^H, and ^13^C NMR analytical techniques (ESI†). X-ray diffraction was employed for those compounds where a block crystal could be isolated. Here, the characterization of compound 6 is presented in detail. In the FTIR spectrum, characteristic vibrations of the oxazinone are observed at 1717 cm^−1^ for the carbonyl (C=O), 1613 cm^−1^ for the imine C=N, 1509 cm^−1^ for the N–O stretching, and 1117 cm^−1^ for the C–O stretching. Vibrations derived from the aromatic rings and their substituents were observed at 3032 cm^−1^ for the aromatic C=C–H stretching and 2916 cm^−1^ for the aliphatic C–H stretching. The MS(EI) spectrum shows the molecular ion [M^+^] at 277 *m*/*z* and the base peak (BP) at 219 *m*/*z*. The BP was generated by the contraction of the heterocycle ring due to the loss of CO and NO (*M* = −58 *m*/*z*) and the aromatic beta cleavage to generate a 116 *m*/*z* fragment. Additionally, the formation of the tropylium ion at 91 *m*/*z* was observed.

**Fig. 2:**
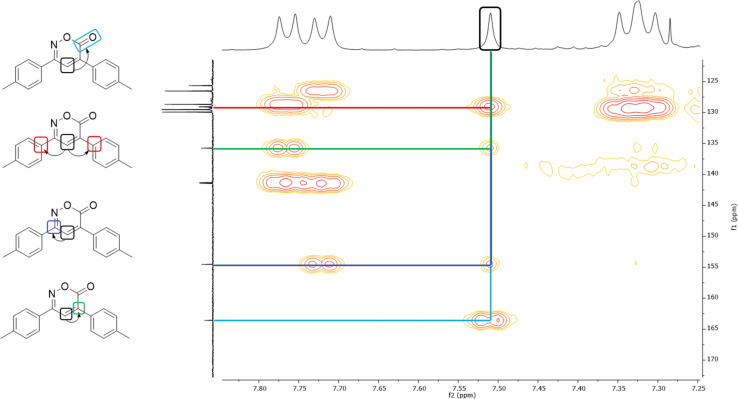
HMBC of 6*H*-1,2-oxazin-6-one 6.

**Fig. 3:**
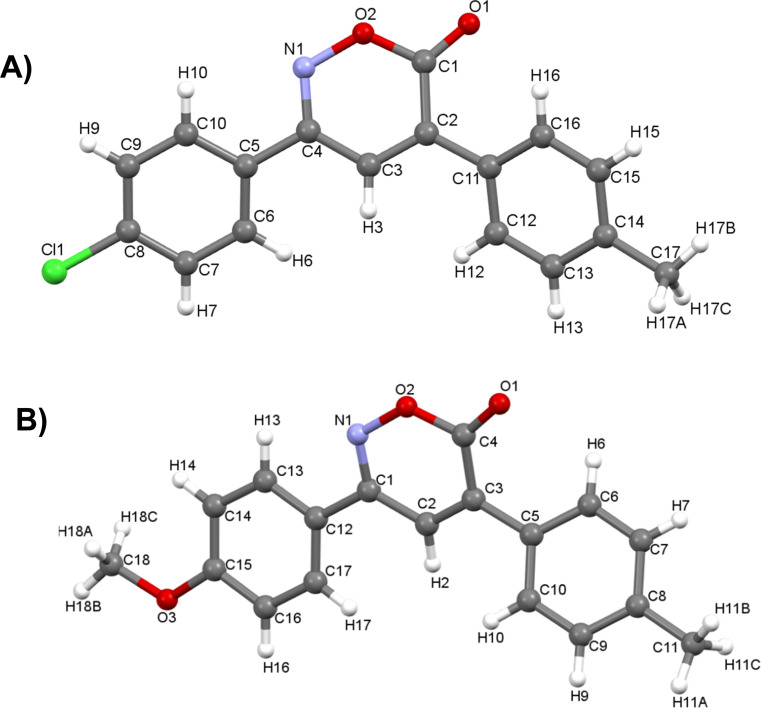
Crystal structures of 6*H*-1,2-oxazin-6-one (A) 1 and (B) 7.

In the ^1^H NMR spectrum, a singlet at 7.51 ppm corresponding to the lone hydrogen present in the heterocycle confirms the formation of the oxazinone. Additionally, the aromatic signals of the two A_2_B_2_ systems are observed in the range of 7.77–7.31 ppm, integrating for eight hydrogens. These signals appear as a doublet at 7.76 ppm with *J* = 7.9 Hz, integrating for two hydrogens, a doublet at 7.72 ppm with *J* = 7.9 Hz, integrating for two hydrogens, and two overlapping doublets at 7.33 ppm, resembling a pseudo-triplet, integrating for the remaining four hydrogens. Finally, singlets at 2.45 and 2.44 ppm are observed, each integrating three hydrogens from the two methyl groups. The ^13^C NMR spectrum shows indicative signals of the oxazinone formation; carbons at 163.6, 154.5, 135.8, and 125.7 ppm correspond to the carbonyl, imine, quaternary carbon, and methine, respectively. The aromatic carbons of the two A_2_B_2_ systems are observed at 129.9, 129.5, 128.7, 126.5, 141.4, 141.3, 129.1, and 129.0 ppm. Finally, signals for the methyl groups are observed at 21.44 and 21.42 ppm. As a complementary study to assign signals unequivocally, two-dimensional HMBC was performed, taking the hydrogen of the 6*H*-1,2-oxazin-6-one as the reference signal, located at 7.51 ppm in the hydrogen spectrum. This signal correlates at three bonds with the carbonyl signal at 163.6 ppm and with the quaternary carbons of the aromatic systems at 129.1 and 129.0 ppm. It also correlates with two bond signals: one at 154.5 ppm of the imine carbon and the second at 135.8 ppm of the quaternary carbon of the heterocycle (Fig. 2). Among the twelve synthesized 6*H*-1,2-oxazin-6-ones, two block crystals corresponding to compounds 1 and 7 were successfully isolated, enabling X-ray crystallographic analyses. These crystals belong to the monoclinic system with the *P*2_1_/*c* space group, with R-factor percentages of 4.51 and 5.66%, respectively (Fig. 3, Tables S3 and S4†).

### Biology

In the antibacterial assay with the 12 6*H*-1,2-oxazin-6-ones, six compounds inhibited the bacterial growth with MICs ranging from 200 μg mL^−1^ to 3.125 μg mL^−1^. Compounds 1 and 4 were the most active with MICs similar to or lower than control antibiotics (Tables 3, S5 and S6†). Compound 1 (R_1_ = 4-Cl, R_2_ = 4-Me) had the highest activity against three Gram-positive strains (ATCC 43300, MRSA 4, MRSA 5) with MIC of 3.125 μg mL^−1^ but failed to inhibit the growth of Gram-negative *E. coli* 25 922, suggesting that 1 showed selectivity against Gram-positive bacteria as Linezolid. On the other hand, oxazinones 4 (R_1_ = 4-Me, R_2_ = 4-Cl) and 8 (R_1_ = 4-Me, R_2_ = 4-OMe) were active against all five bacteria with MICs ranging from 12.5 μg mL^−1^ to 25 μg mL^−1^, affecting both Gram-positive and Gram-negative bacteria. Although the concentrations at which the bacteria were sensitive are higher than those for the control drugs, compound 4 presented an activity not shown by Linezolid.^26^

**Table 3:** Determination of MIC's of 6*H*-1,2-oxazin-6-ones^a^

6*H*-1,2-oxazin-6-ones			43 300	SARM 3	SARM 4	SARM 5	25 922
	R_1_	R_2_					
1	4-Cl	4-Me	3.125	—	3.125	3.125	—
2	4-H	4-Me	200	200	200	200	200
3	4-Cl	4-Cl	100	—	—	—	—
4	4-Me	4-Cl	12.5	12.5	12.5	12.5	12.5
6	4-Me	4-Me	50	50	—	50	—
8	4-Me	4-OMe	25	25	25	25	25
Gentamicin			2	1	2	2	1
Linezolid			4	4	8	8	—
Trimethoprim			1	1	2	2	—

a MRSA ATCC 43300, clinical Isolates MRSA 3, MRSA 4, MRSA5, *E. coli* ATCC 25922. (—): no activity.

Finally, compound 6 (R_1_ = 4-Me, R_2_ = 4-Me) showed activity against three Gram-positive bacteria (ATCC 43300, MRSA 3, MRSA 5) with MIC of 50 μg mL^−1^. Thus, the *in vitro* antibacterial results suggest the potential of these oxazinones in treating infections caused by multi-resistant *Staphylococcus aureus*, a pathogen frequently isolated worldwide that causes a wide range of clinical manifestations.^17^

Research on the antibacterial activity of 1,2-oxazinones is limited; most studies focus on 1,3- or 1,4-oxazinones, showing MIC values ranging from 8 to 250 μg mL^−1^.^5,6,25,60^ Only three studies have documented 1,2-oxazinones and their activity against bacteria. Two of these studies used the Kirby–Bauer method with inhibition zones of 10 to 25 mm, while the third study employed the broth microdilution method and determined MIC values of 0.25 to 0.5 μg mL^−1^, which are lower than those found for 1 and 4. Additionally, the trend against Gram-positive bacteria was maintained.^11,21,24^ Another report described the synthesis and antibacterial evaluation of β-cyanoketones, finding MIC values of 200 μg mL^−1^ against Gram-positive bacteria.^32^ Subsequently, upon conversion into oxazinones, these MIC values decreased to as low as 3.12 μg mL^−1^, emphasizing the importance of the heterocycle in enhancing antibacterial activity.

The structure–activity analysis of the most active compounds (1 and 4) showed that similar substituents, –Cl and –Me, are positioned at opposing locations in the aromatic rings (R_1_ and R_2_). This characteristic results in a potency of oxazinone 1 being four times greater than oxazinone 4. Hence, the orientation of these substituents on the aromatic rings is crucial for interaction with their target. Furthermore, combining an electron-donating group and an electron-withdrawing group on each ring is essential for achieving the antibacterial effect. This characteristic is not observed in compounds 9 and 10, where one contains an electron-donating group (–OMe) and the other an electron-withdrawing group (–Cl), therefore, the presence of the methoxy substituent in these compounds led to the absence of activity, even obtaining favorable scores through docking. On the other hand, the antibacterial activity of oxazinones with two mixed electron-donating or electron-withdrawing groups is decreased or lost. A similar effect is observed when there is no substituent on the rings.

**Fig. 4:**
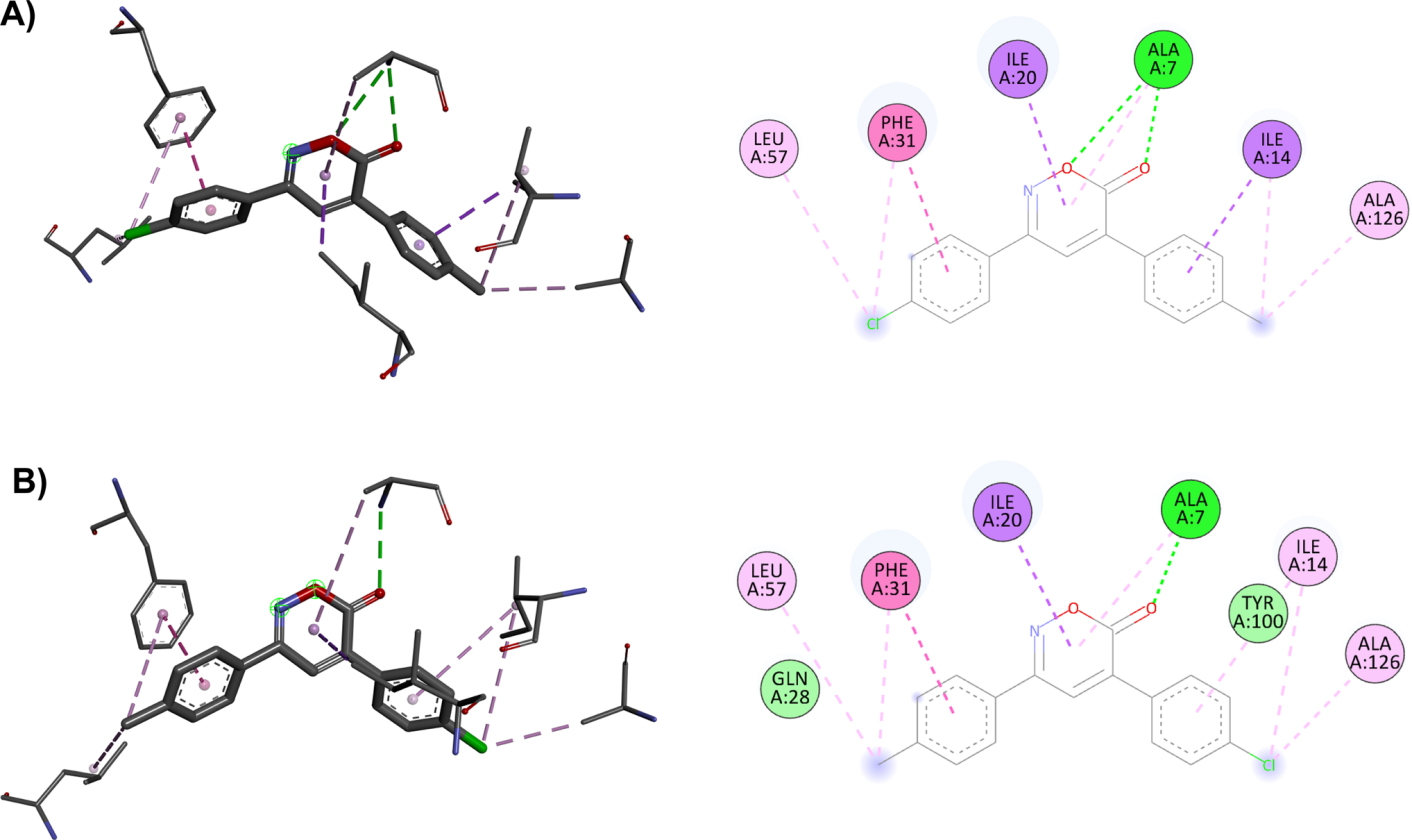
2D and 3D interaction complex of 6*H*-1,2-oxazin-6-ones (A) 1 and (B) 4 with DHFR.

The docking in the DHFR shows differences in the number of interactions due to changes in the substituents in opposing positions of the aromatic rings, resulting in different score values: 9.0 kcal mol^−1^ for compound 1 and 8.7 kcal mol^−1^ for compound 4. In the modeling of compound 1, the two hydrogen bond interactions of the oxazinone with Ala7 are crucial for its effect, whereas compound 4 has only one hydrogen bond. Ultimately, these hydrogen bond interactions with DHFR are essential for the activity of the oxazinones (Fig. 4).

Finally, the results generated through molecular dynamics study indicate that compound 4 exhibits high stability in complex with the DHFR enzyme over a 100 ns simulation. It is suspected that the mechanism of action of the oxazinones follows this model, leading to a more spontaneous interaction for compound 1. However, this interaction may not be stable enough, as in the case of compound 4, to exert its effect against various bacteria. For instance, with *E. coli*, due to different defense mechanisms and the inherent nature of the bacteria, compound 1 may not reach its target site, or the interaction time at the target site may not be sufficient to generate the activity observed in comparison to compounds 4 and 8.

**Fig. 5:**
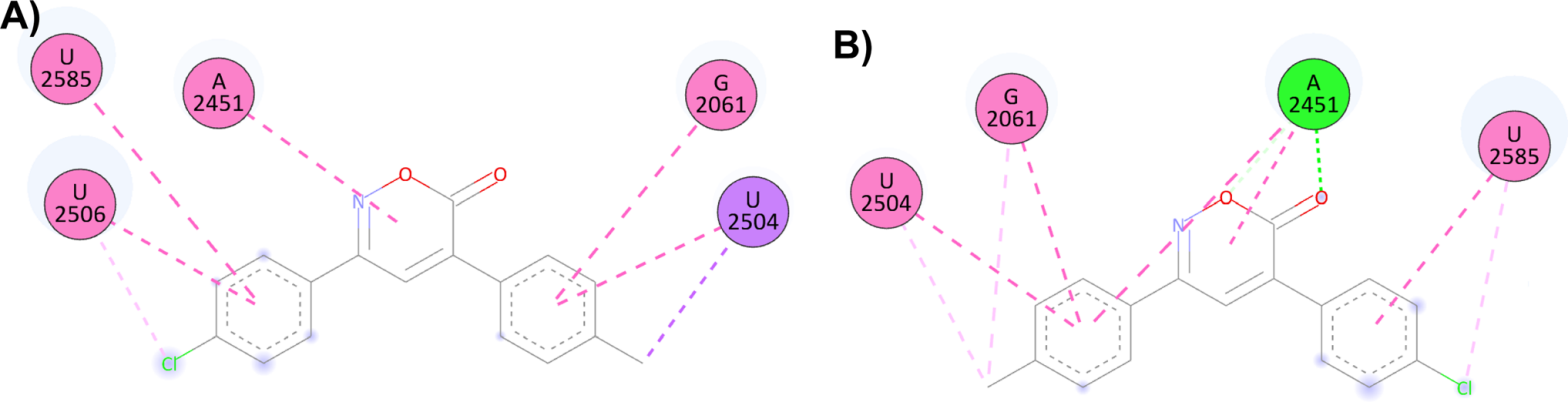
2D and 3D interaction complex of 6*H*-1,2-oxazin-6-ones (A) 1 and (B) 4 in the PTC.

On the other hand, the docking results with the PTC show that compound 4 has more interactions than compound 1, possibly explaining the broader antibacterial spectra of 4. As previously mentioned, 4 (score = −8.5 kcal mol^−1^) is one of the oxazinones with the highest interactions with the PTC. Compared to compound 1 (score = −8.0 kcal mol^−1^), compound 4 formed two hydrogen bonds with A2451 and more hydrophobic interactions. Similarly, compound 8 also showed a better score (−8.3 kcal mol^−1^) than compound 1 in this model and inhibited the growth of all five bacteria (Fig. 5). The results of this study shed some light on the mechanism of the antibacterial activity of oxazinones, and future experiments are necessary to clarify this situation. However, molecular dynamics simulations of the oxazinones with DHFR provide a broader understanding of their interactions and stability.

### LD_50_ toxicity prediction

The predicted LD_50_ values ranged from 180 to 3880 mg kg^−1^. The compounds were classified based on the six categories of the GHS system (Globally Harmonized System of Classification and Labeling of Chemicals, rev. 8) as follows: category I, LD_50_ ≤ 5 mg kg; category II, 5 < LD_50_ ≤ 50 mg kg; category III, 50 < LD_50_ ≤ 300 mg kg; category IV, 300 < LD_50_ ≤ 2000 mg kg; category V, 2000 < LD_50_ ≤ 5000 mg kg; and category VI, LD_50_ > 5000 mg kg^−1^. Therefore, the software classified the toxicity of 6*H*-1,2-oxazin-6-ones into category IV (400–1000 mg kg^−1^), which encompasses a very high concentration range compared to the determined MICs, thus identifying the compounds as non-toxic.^51,52^

### Toxicity evaluation in the *Artemia salina* model

The six 6*H*-1,2-oxazin-6-ones that show antibacterial activity, did not present adverse effects on the development and viability of phototropic nauplii of *A. salina* at 200 μg mL^−1^. Additionally, Tween 80 at 10% also shows no negative effects on the viability and development of *A. salina*. Therefore, the oxazinones are deemed harmless to *A. salina* (LD_50_ > 200 μg mL^−1^), supporting the predictions of the *in silico* studies (Table S7†).

## Conclusions

This study employed a new synthetic route for preparing 12 6*H*-1,2-oxazin-6-ones without additives, with yields ranging from moderate to high (55–88%). Future efforts will focus on refining the reaction conditions to enhance yields. Notably, the antibacterial activity of the synthesized oxazinones proved clinically relevant when tested against resistant bacteria, particularly MRSA, showing even better activity than Linezolid, a commonly prescribed antibiotic for such infections (MIC of 1 = 3.125 μg mL^−1^). The active compounds were also not toxic in both *in silico* and *in vitro* assays. Hence, these selected oxazinones are promising for developing new antibiotics targeting multidrug-resistant bacteria infections.

## Data availability

The data supporting this article have been included as part of the ESI†

## Author contributions

Eleazar Alcántar-Zavala investigation, conceptualization, methodology, formal analysis, writing – original draf. Francisco Delgado-Vargas: formal analysis, funding acquisition, resources, review & editing. Fabricio Marín-González: investigation, methodology. Grabriela López-Angulo: resources. Hugo Enrique Aguirre-Madrigal: investigation, methodology. Adrián Ochoa-Terán: resources, review & editing. Gibrán Rodríguez-Vega: resources, software; visualization, writing. Gerardo Aguirre-Hernández: resources. Julio Montes-Avila: conceptualization, formal analysis, funding acquisition, writing – review & editing.

## Conflicts of interest

The authors declare that they have no known competing financial interests or personal relationships that could have appeared to influence the work reported in this paper.

## Supplementary Material

RA-014-D4RA04220D-s001

RA-014-D4RA04220D-s002
