# Supplementary material for: Design, synthesis, and exploration of antibacterial activity of 6H-1,2-oxazin-6-ones
Source: RSC Adv. 2024 Jul 29;14(33):23828–39. doi: 10.1039/d4ra04220d (PMC11285021; doi:10.1039/d4ra04220d)
Supplement: RA-014-D4RA04220D-s001 [file RA-014-D4RA04220D-s001.pdf]

Supplementary Information (SI) for RSC Advances.
This journal is © The Royal Society of Chemistry 2024

Supplementary Information

Design, Synthesis and Exploration of Antibacterial Activity of 6*H*-1,2-oxazin-6-ones

Eleazar Alcántar-Zavala,[a] Francisco Delgado-Vargas,[b] Fabricio Marín-González,[a] Gabriela López Angulo,[a] Hugo Enrique Aguirre-Madrigal,[b] Adrián Ochoa-Terán,[c] Gibrán Rodríguez-Vega,[d] Gerardo Aguirre-Hernández,[c] Julio Montes-Avila*[b]

[a]Facultad de Ciencias Químico-Biológicas, Universidad Autónoma de Sinaloa, Culiacán, Sinaloa, 80010 México

[b]Programa de Posgrado en Ciencias Biomédicas, Facultad de Ciencias Químico-Biológicas, Universidad Autónoma de Sinaloa, Culiacán, Sinaloa, 80010 México. E-mail: jmontes@uas.edu.mx

[c]Centro de Graduados e Investigación en Química, Tecnológico Nacional de México/Instituto Tecnológico de Tijuana, Tijuana, Baja California, 22444 México

[d]Unidad Académica de Ciencias Químico-Biológicas y Farmacéuticas, Universidad Autonóma de Nayarit, Tepic, Nayarit, 63155 México

Table S1. Docking scores of designed oxazinones with DHFR enzyme and the PTC.

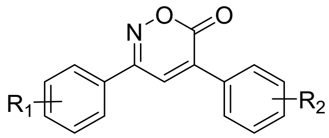

			DHFR	PTC
**Oxazinones**	**R_1**	**R_2**	**Score (ΔG, kcal / mol)**	**Score (ΔG, kcal / mol)**
**16**	4-OH	4-OH	-8.5	-8.2
**17**	-H	4-OH	-8.5	-8.2
**18**	4-OH	-H	-8.5	-8.1
**19**	3-OH	-H	-8.4	-8.4
**20**	3-OH	3-OH	-8.3	-8.7
**21**	4-OH	3,4-diOH	-8.5	-8.8
**22**	4-OH	4-OMe	-8.6	-8.7
**23**	4-OMe	4-OMe	-8.6	-8.5
**24**	4-NH_2	4-OMe	-8.6	-8.3
**25**	4-NH_2	2-Cl	-8.1	-7.9
**26**	4-NH_2	4-OH	-8.5	-8.3
**27**	4-OH	2-Cl	-8.5	-7.9
**28**	4-NH_2	2-F	-8.6	-8.1
**29**	3-OH	4-Me	-8.7	-8.6
**30**	4-OH	4-Me	-8.9	-8.4
**Linezolid**				-8.9
**Trimethoprim**			-6.7	

Table S2. Prediction of the pharmacokinetic parameters (ADME properties) of the 6*H*-1,2-oxazin-6-ones selected for docking studies.

Oxazinones	MW	NHA	NHD	NRB	TPSA	LogP	LogS	SW	Ro5
**1**	297,74	3	0	2	43,1	4,11	-5	2,99E-03	0
**2**	263,29	3	0	2	43,1	3,59	-4,42	1,00E-02	0
**3**	318,15	3	0	2	43,1	4,31	-5,29	1,61E-03	0
**4**	297,74	3	0	2	43,1	4,12	-5	2,99E-03	0
**5**	283,71	3	0	2	43,1	3,78	-4,72	5,46E-03	0
**6**	277,32	3	0	2	43,1	3,92	-4,71	5,44E-03	0
**7**	293,32	4	0	3	52,33	3,57	-4,47	1,00E-02	0
**8**	293,32	4	0	3	52,33	3,58	-4,47	1,00E-02	0
**9**	313,74	4	0	3	52,33	3,76	-4,76	5,49E-03	0
**10**	313,74	4	0	3	52,33	3,76	-4,76	5,49E-03	0
**11**	297,74	3	0	2	43,1	4,12	-5	2,99E-03	0
**12**	318,15	3	0	2	43,1	4,31	-5,29	1,61E-03	0
**13**	293,32	4	0	3	52,33	3,57	-4,47	1,00E-02	0
**14**	313,74	4	0	3	52,33	3,78	-4,76	5,49E-03	0
**15**	293,32	4	0	3	52,33	4,11	-5	2,99E-03	0

MW is the molecular weight (g/mol), NHA is the number of hydrogen bond acceptors, NHD is the number of hydrogen bond acceptors, NRB is the number of rotatable bonds, TPSA is the topological polar surface area, cLogP is the logarithm of the octanol/water partition coefficient, LogS is the logarithm of the solubility in water, SW solubility in water (mg/mL), Ro5 is Lipinski's rule of five.

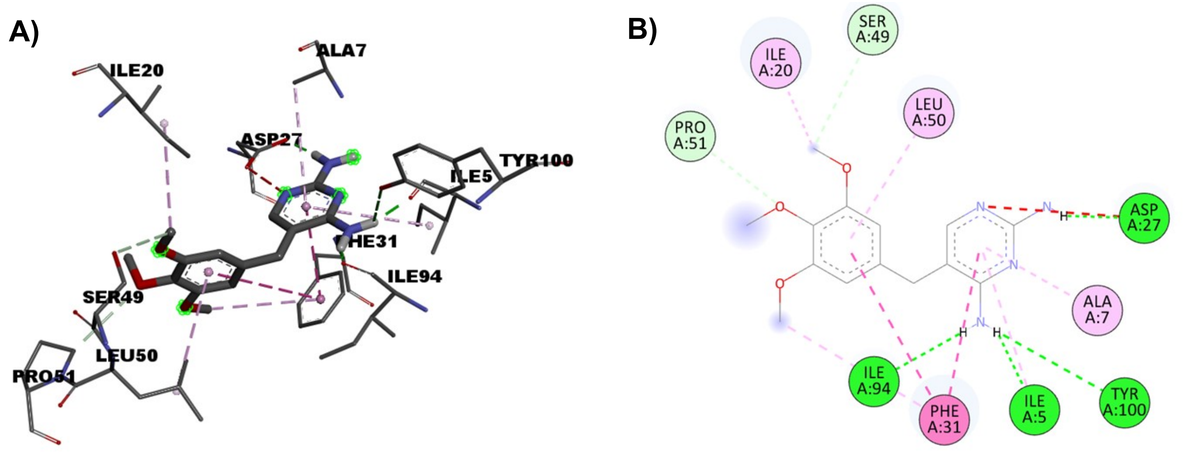

ıre S1. **A**) Trimethoprim in the DHFR binding site; **B**) 2D map of the Trimethoprim ligand in the binding site.

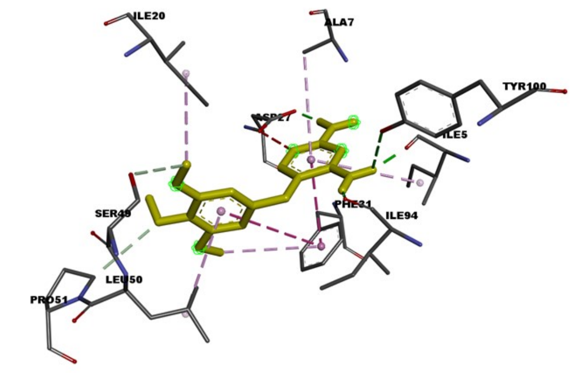

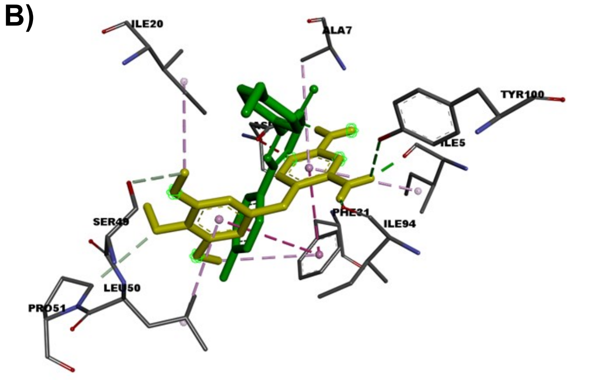

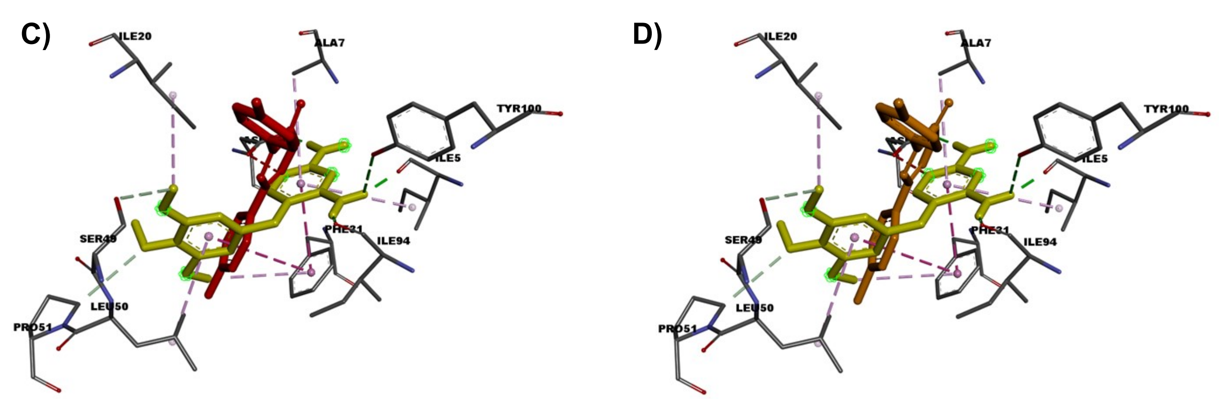

Figure S2. **A)** Trimethoprim (yellow) in the binding site of the DHFR enzyme and

oxazinones **B**) **6** (green), **C**) **8** (red) and **D**) **3** (orange) superimposed.

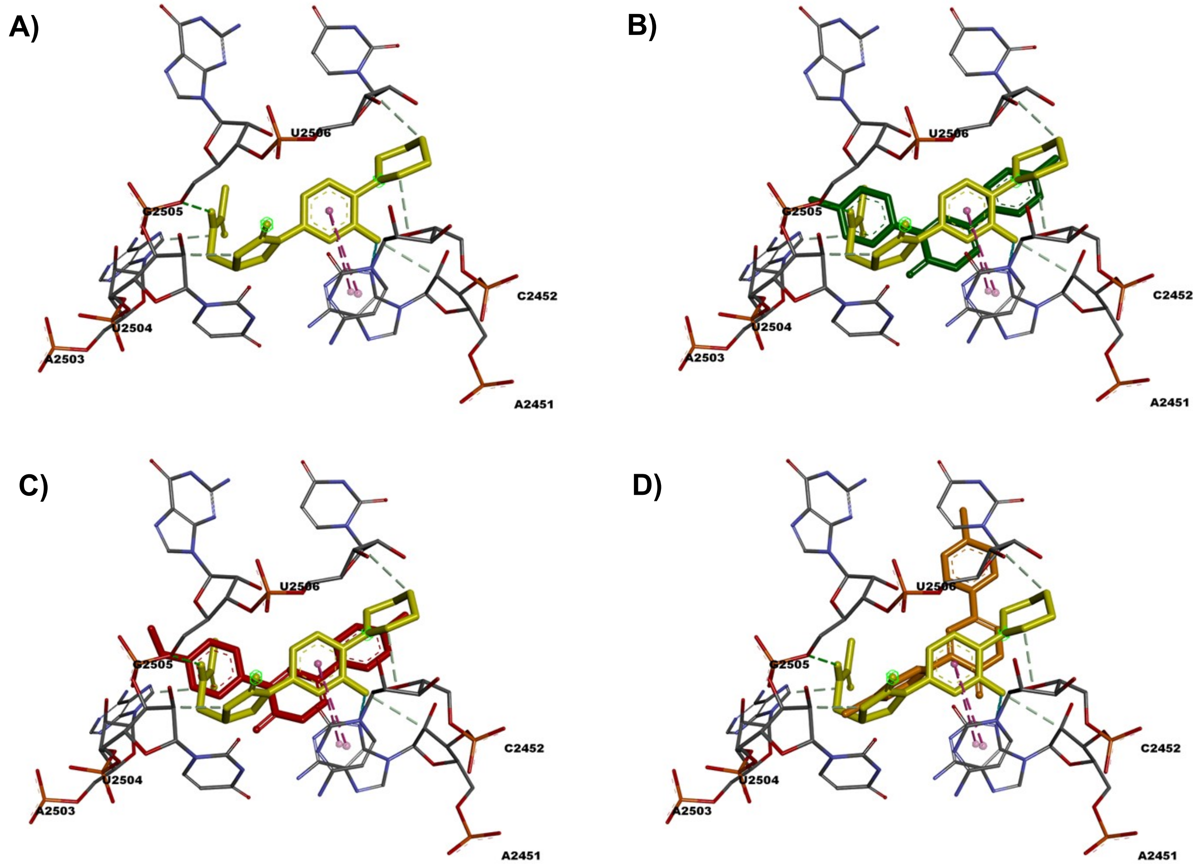

Figure S3. **A**) Linezolid (yellow) in the binding site of the SFT and oxazinones **B**) **6** (green), **C**) **8** (red) and **D**) **3** (orange) superimposed.

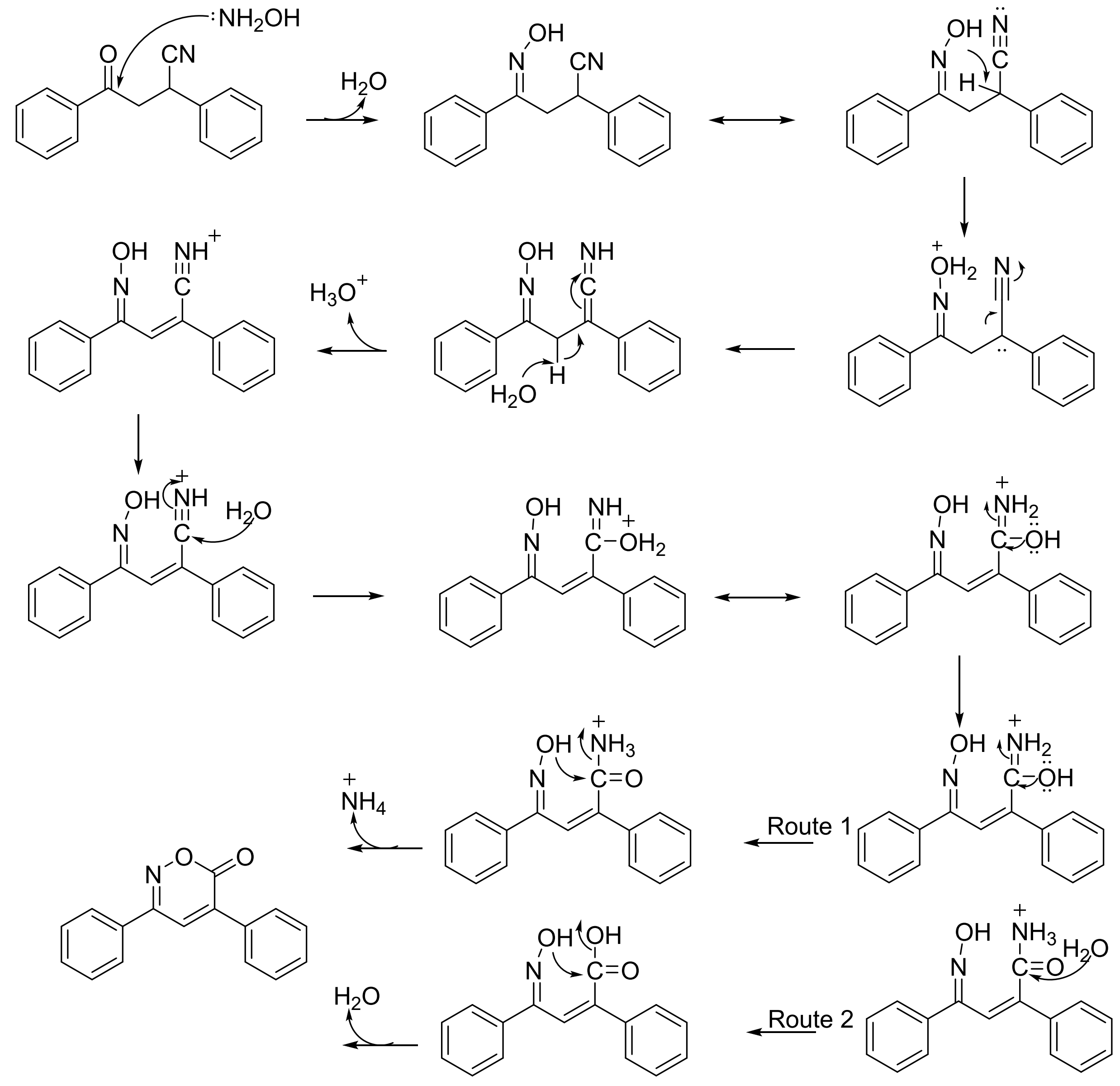

Scheme 1S. Proposed mechanism for the synthesis of 6*H*-1,2-oxazin-6-ones from β-cyanoketones.

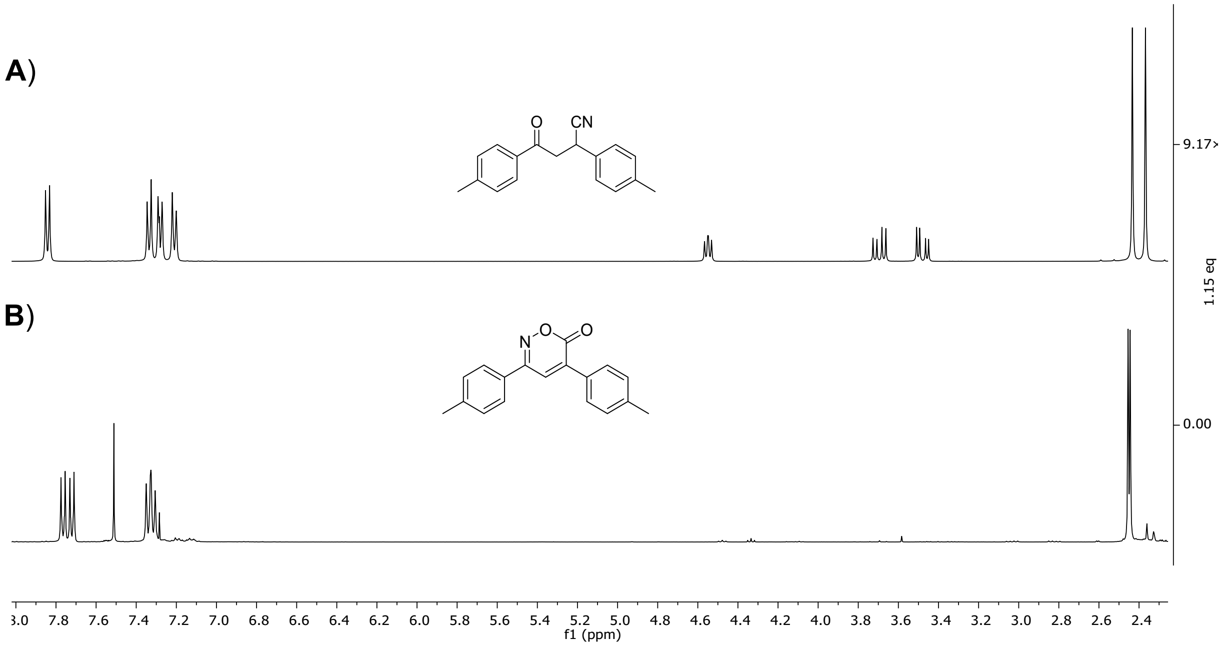

Figure S4. Partial ^{1}H-NMR spectra of β-cyanoketones precursor (**A**) and **6 B**).

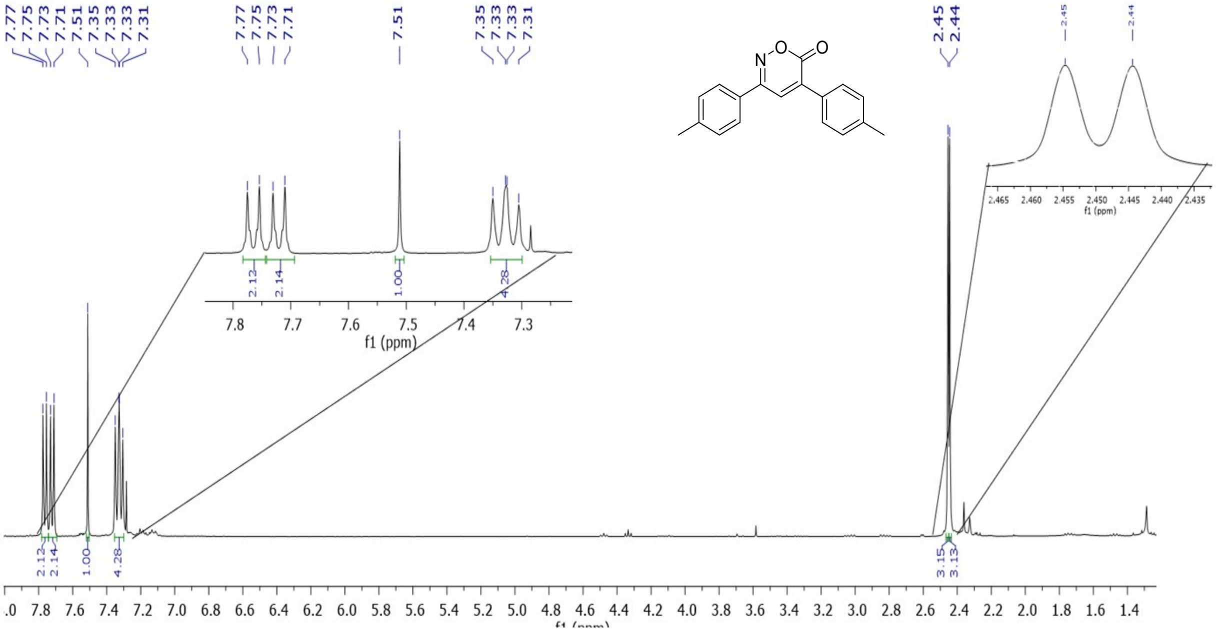

Figure S5. ^{1}H-NMR spectrum of **6** in $CDCl_3$.

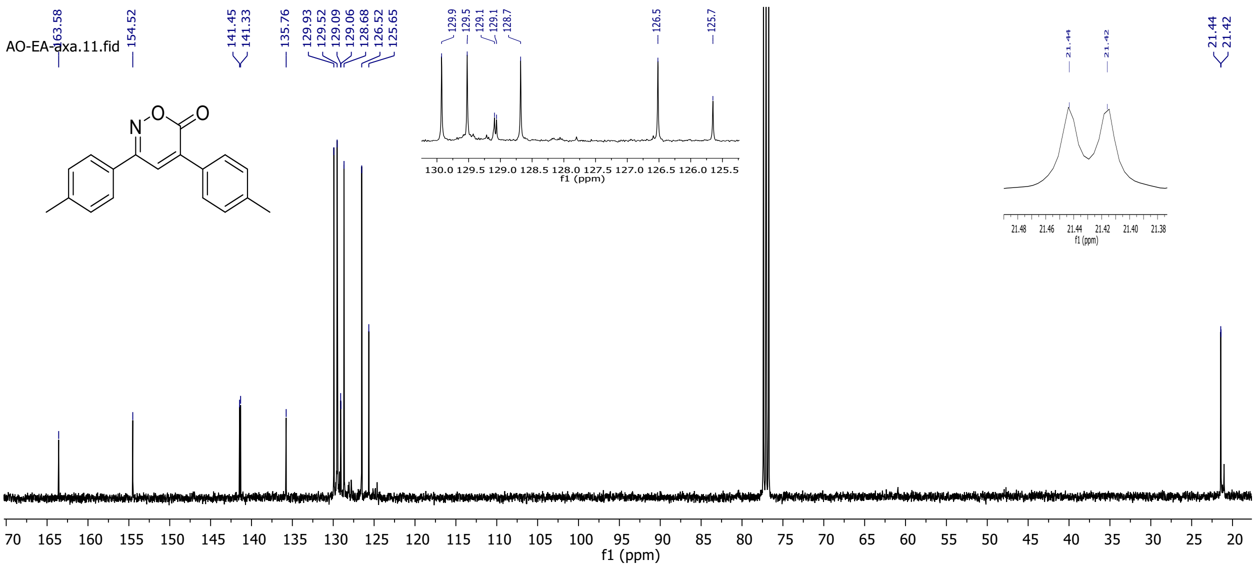

Figure S6. ^{13}C-NMR spectrum of **6** in $CDCl_3$.

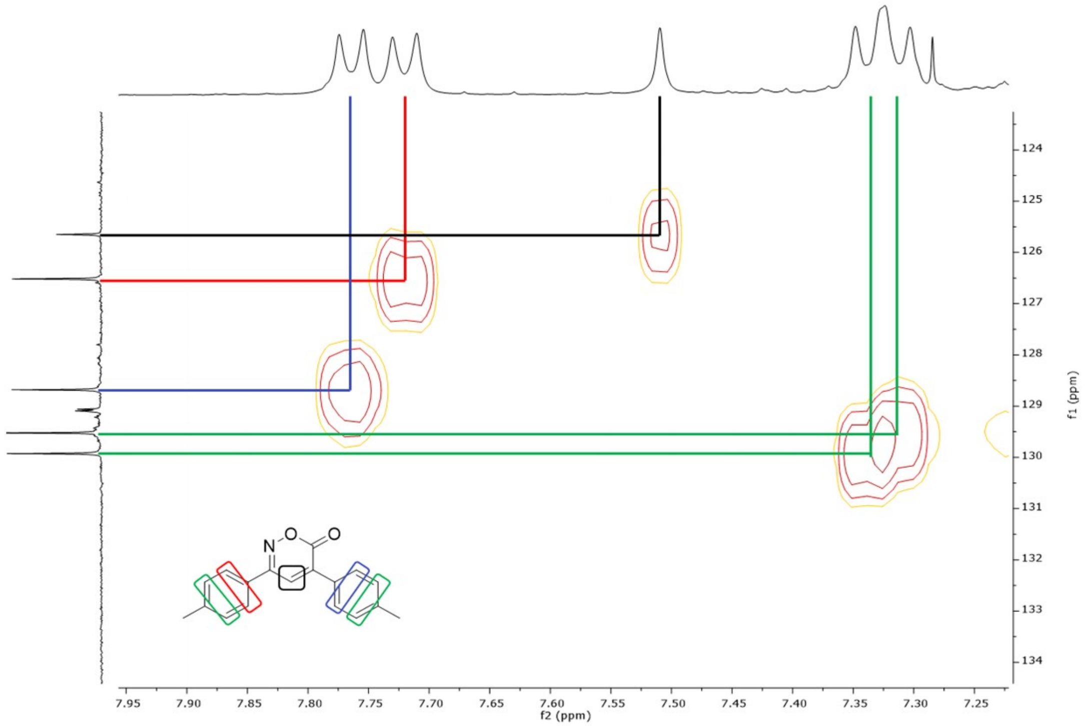

Figure S7. HSQC spectrum of **6**.

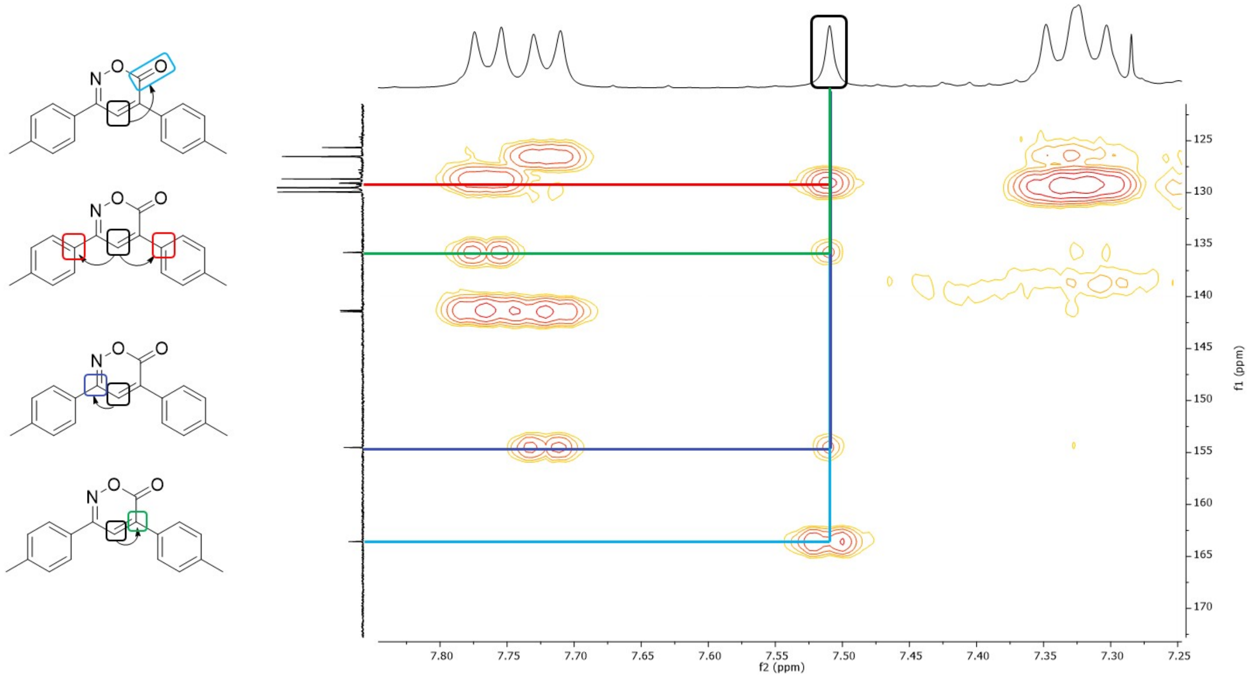

Figure S8. HMBC spectrum of **6 (part 1)**.

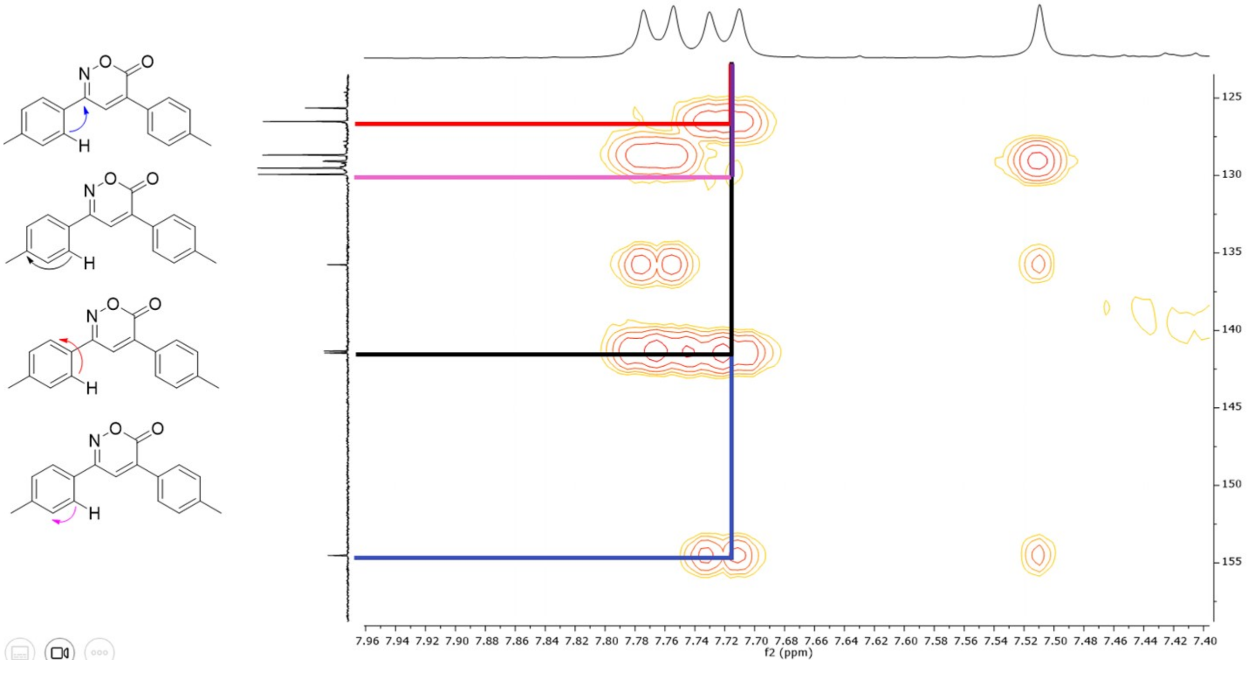

Figure S9. HMBC spectrum of **6 (part 2)**.

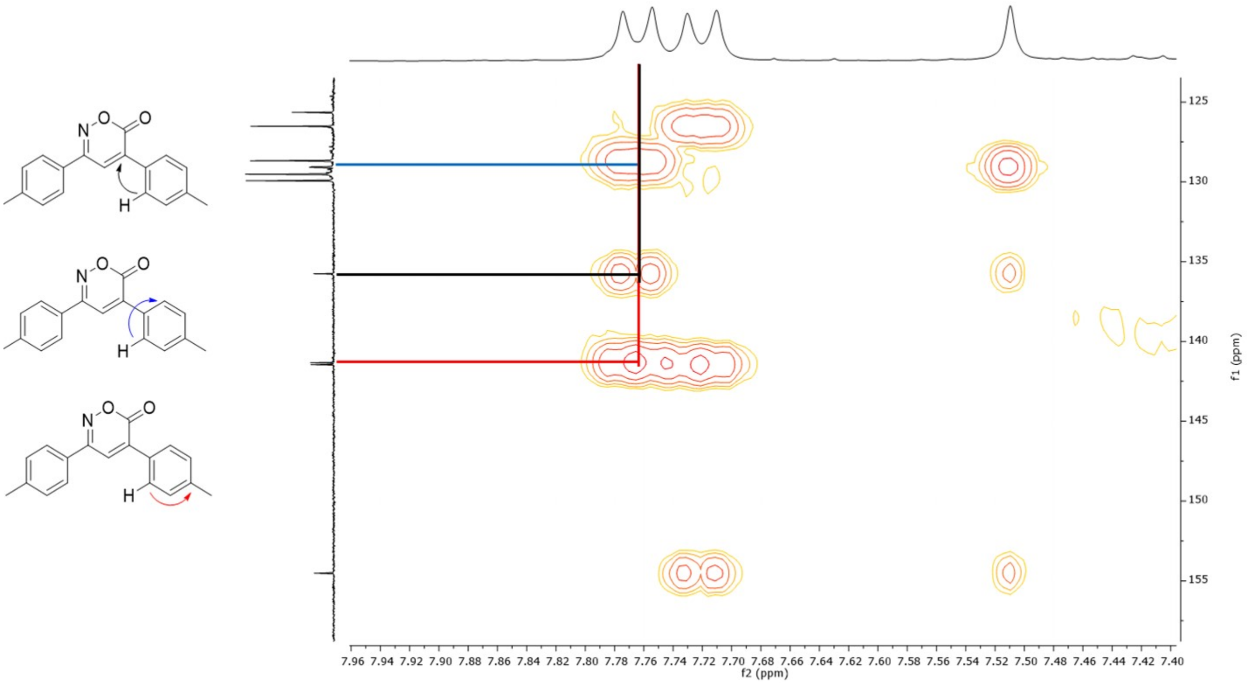

Figure S10. HMBC spectrum of **6 (part 3)**.

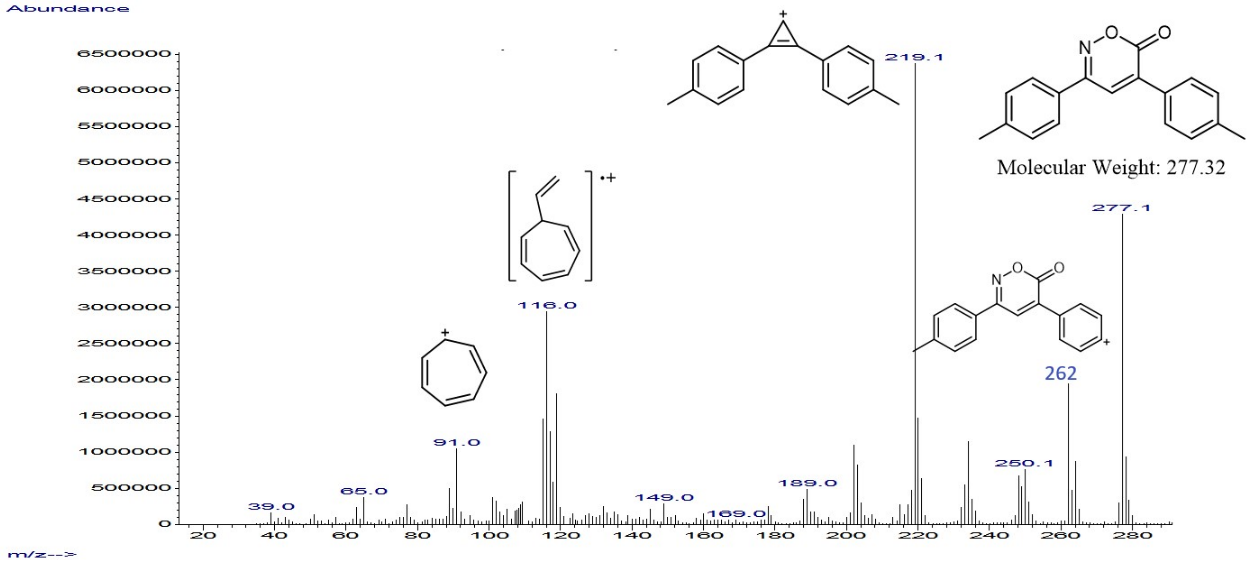

Figure S11. MS(IE) of **6**.

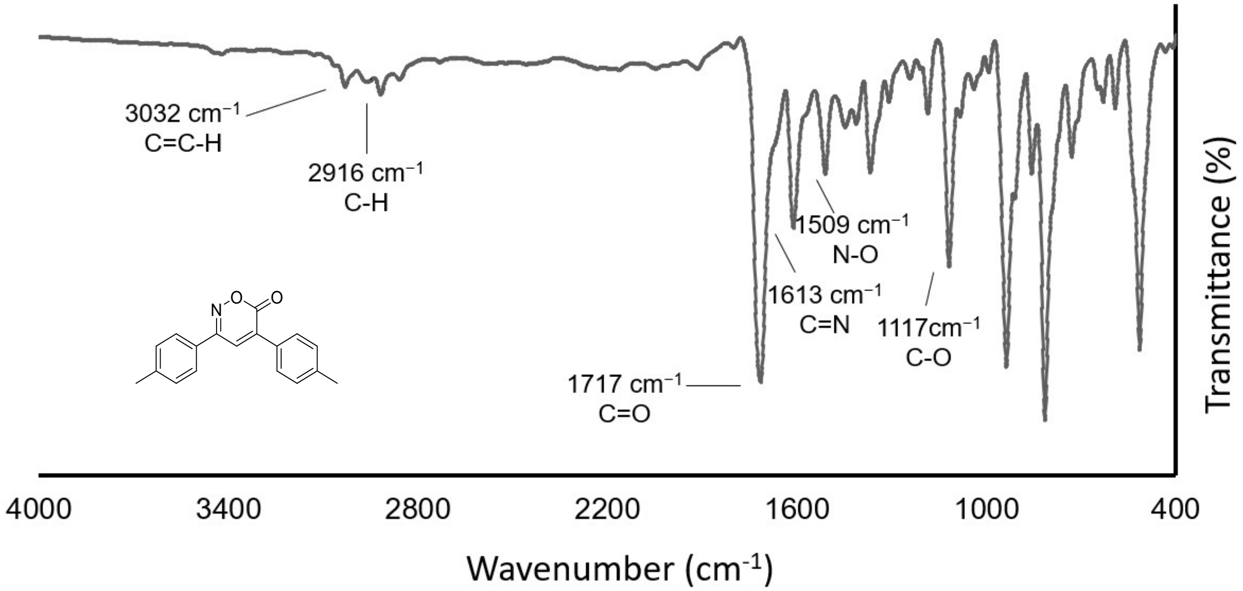

Figure S12. FT-IR of **6**.

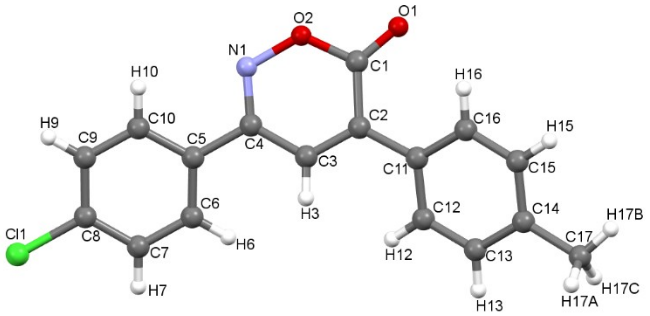

Table S3. Crystal data and structure refinement for EA-20000.

Identification code	**6*H*-1,2-oxazin-6-ones (1)**
Empirical formula	$C_{17}H_{12}ClNO_2$
Formula weight	297.73
Temperature/K	294.1(4)
Crystal system	monoclinic
Space group	$P2_1/c$
a/Å	6.54533(13)
b/Å	29.5464(5)
c/Å	7.4552(2)
α/°	90
β/°	101.691(2)
γ/°	90
Volume/Å^3	1411.85(5)
Z	4
ρ_{calc}g/cm^3	1.401
μ/mm^{-1}	2.424
F(000)	616.0
Crystal size/mm^3	0.183 × 0.067 × 0.054
Radiation	CuKα (λ = 1.54184)
2Θ range for data collection/°	5.982 to 141.54
Index ranges	-8 ≤ h ≤ 8, -35 ≤ k ≤ 36, -8 ≤ l ≤ 8
Reflections collected	18218
Independent reflections	2676 [R_{int} = 0.0423, R_{sigma} = 0.0213]
Data/restraints/parameters	2676/0/191
Goodness-of-fit on F^2	1.036
Final R indexes [I>=2σ (I)]	R_1 = 0.0451, wR_2 = 0.1197
Final R indexes [all data]	R_1 = 0.0554, wR_2 = 0.1273
Largest diff. peak/hole / e Å^{-3}	0.21/-0.21

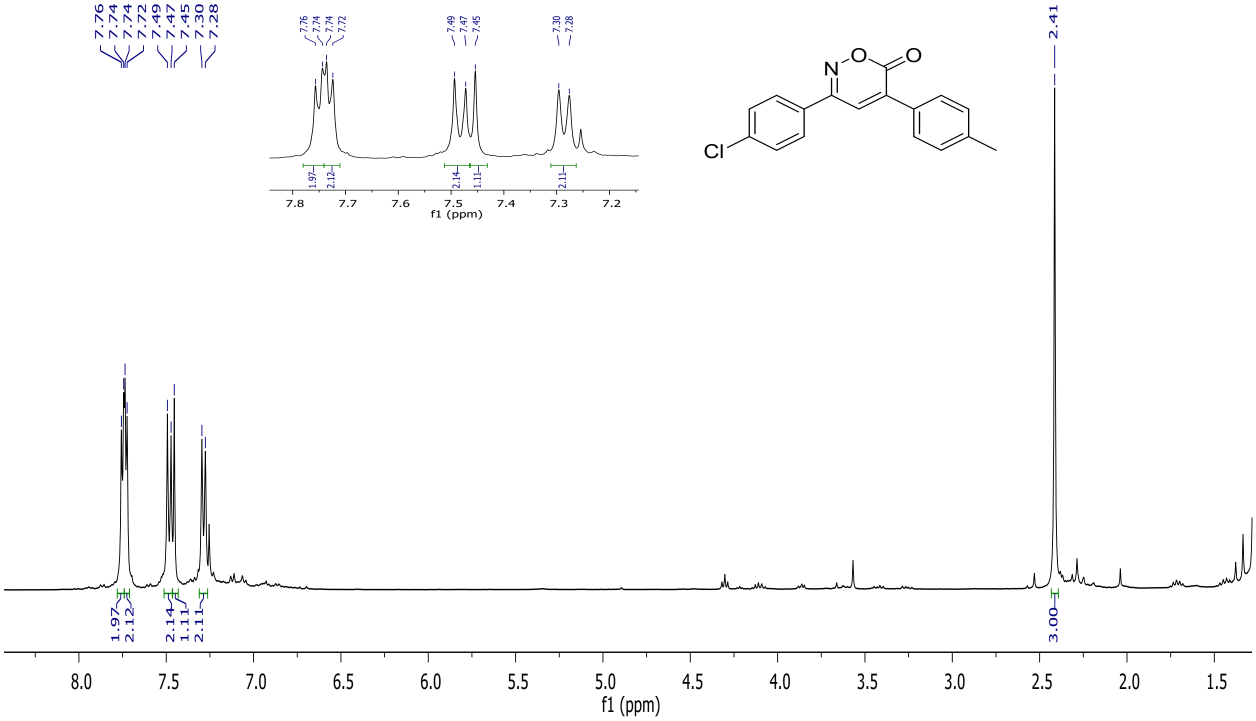

Figure S13. ^{1}H-NMR spectrum of **1** in $CDCl_3$.

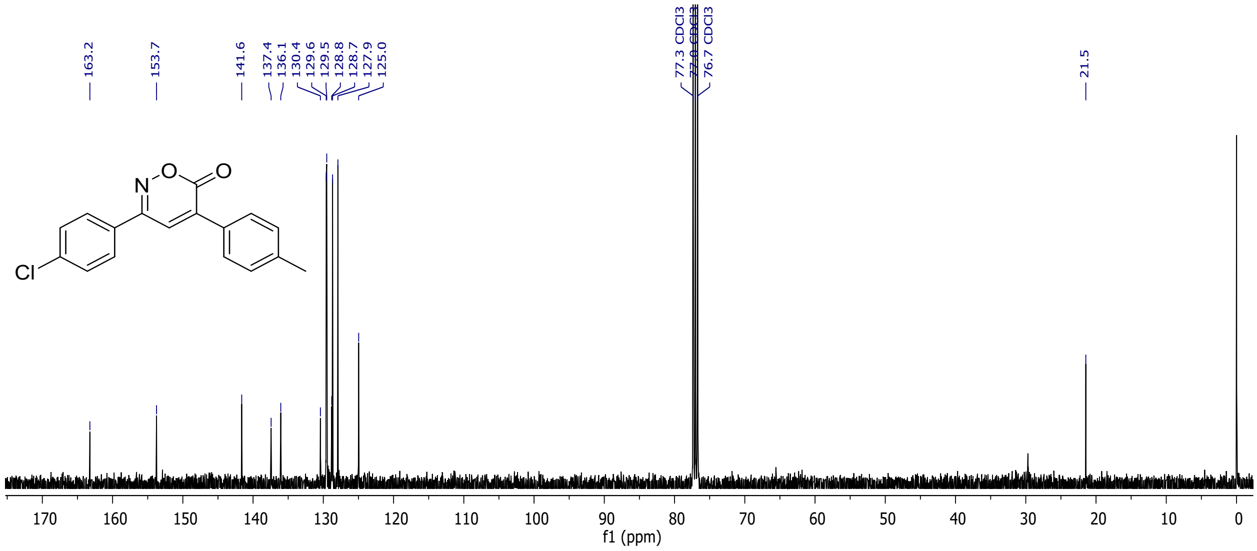

Figure S14. ^{13}C-NMR spectrum of **1** in $CDCl_3$.

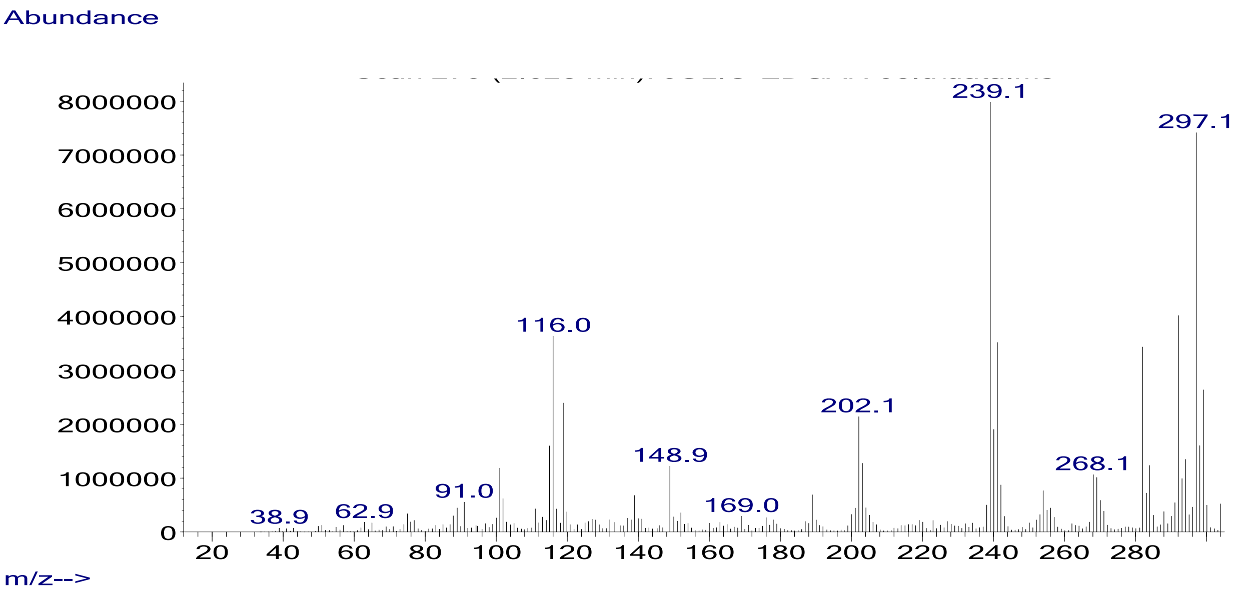

Figure S15. MS(IE) of **1**.

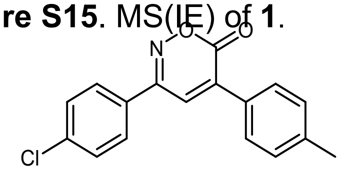

Molecular Weight: 297.74

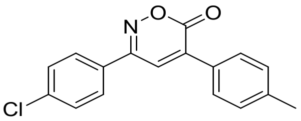

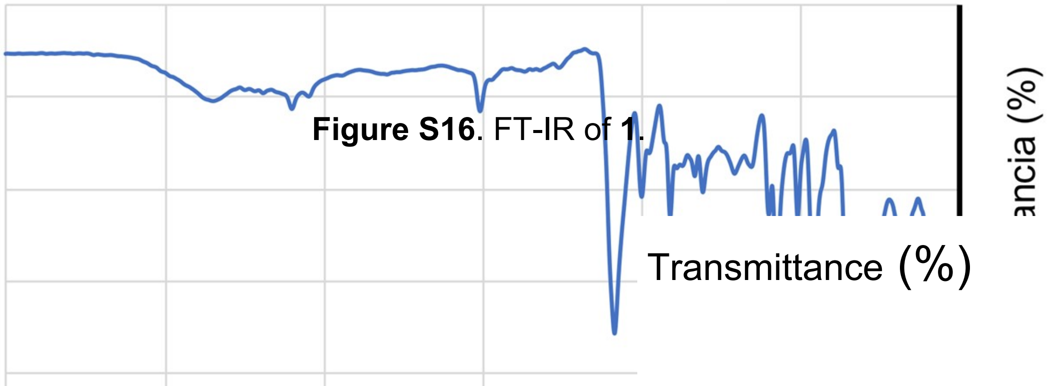

Figure S16. FT-IR of **1**.

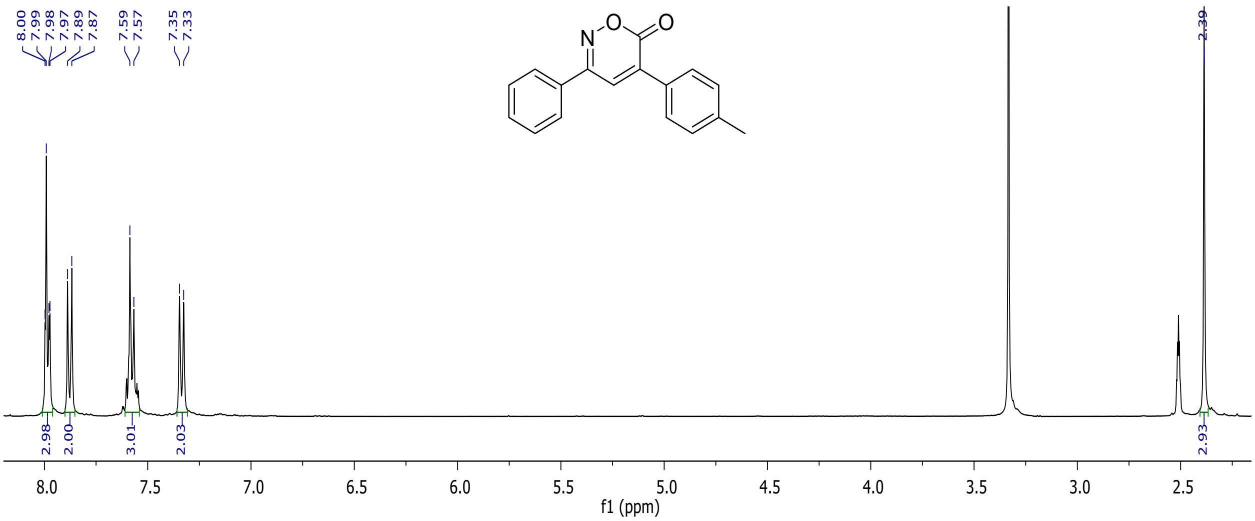

Figure S17. ^{1}H-NMR spectrum of **2** in DMSO-d_6.

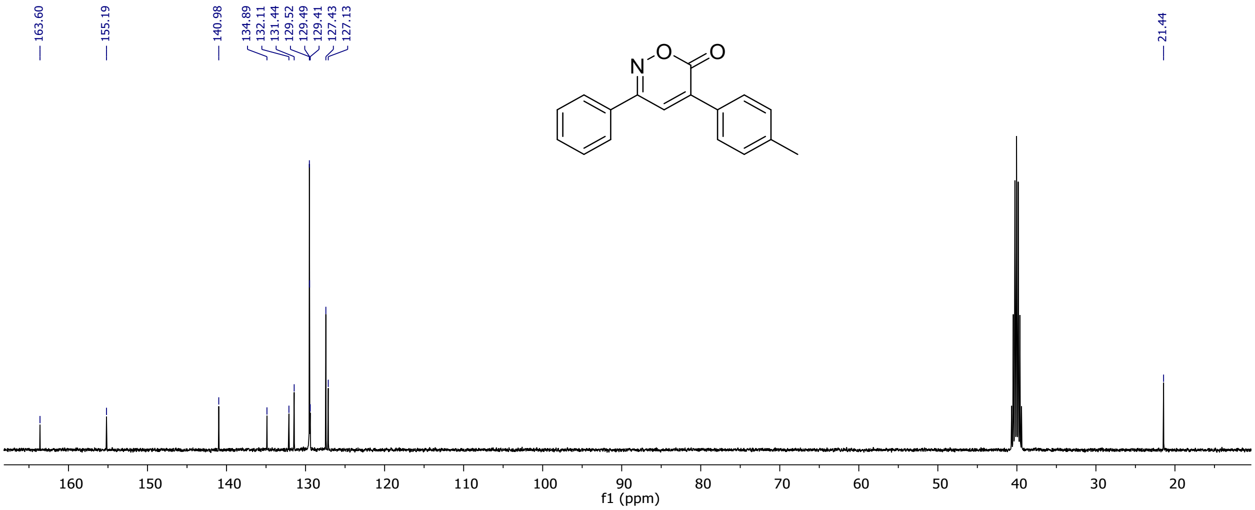

Figure S18. ^{13}C-NMR spectrum of **2** in DMSO-d_6.

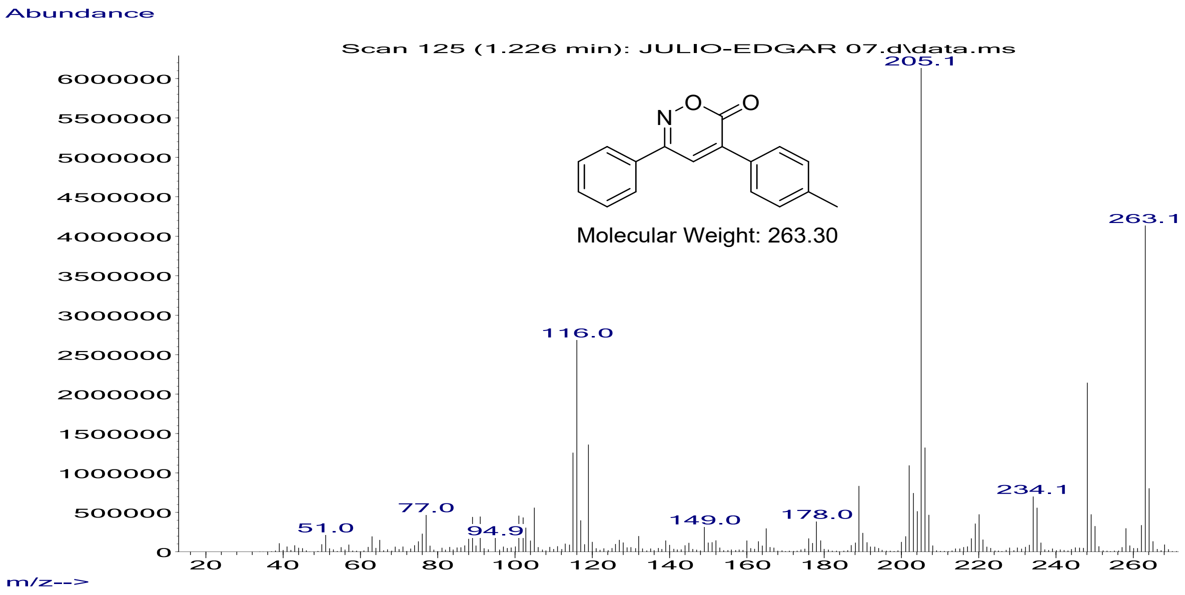

Figure S19. MS(IE) of **2**.

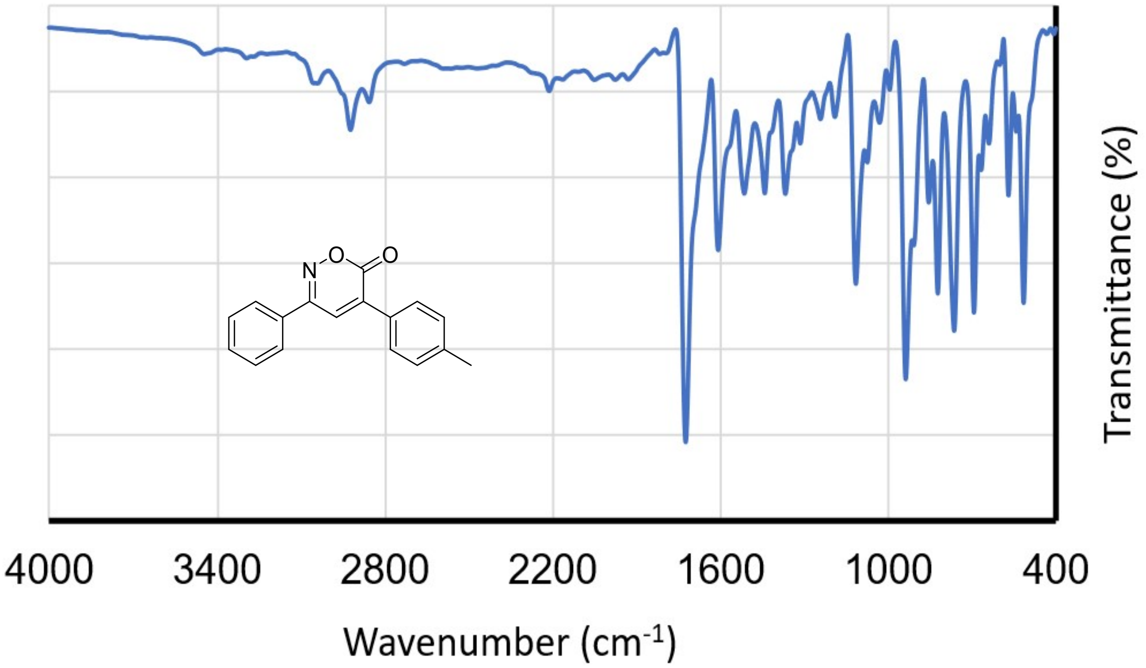

Figure S20. FT-IR of **2**.

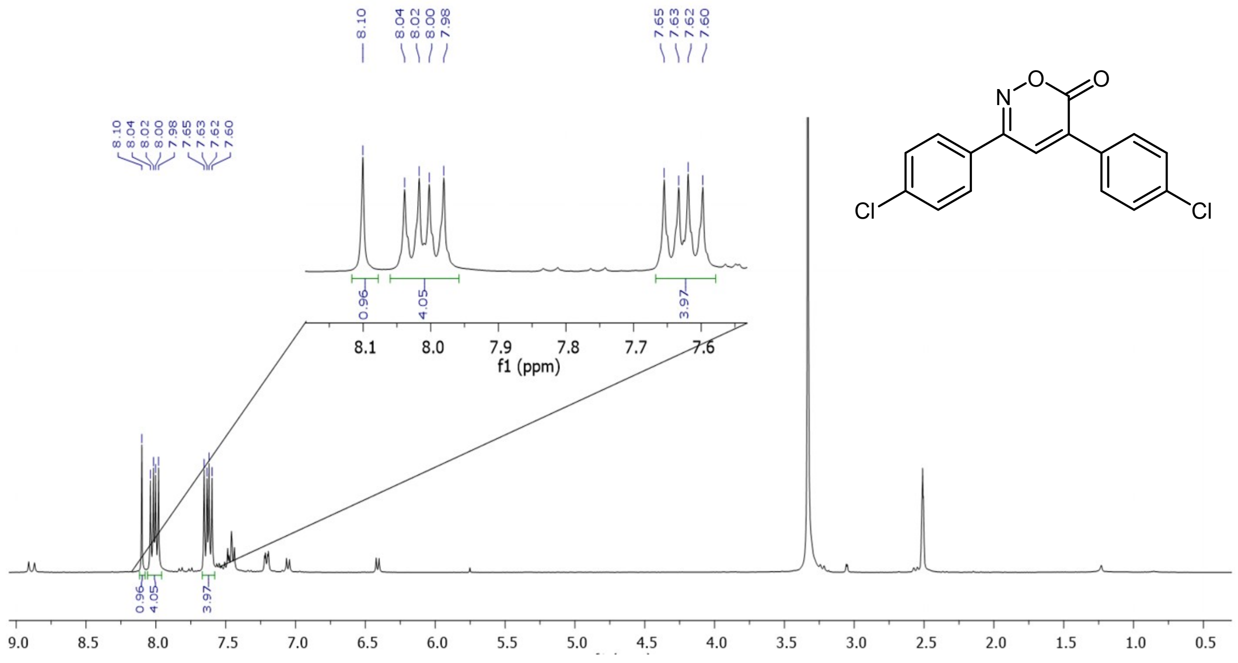

Figure S21. ^{1}H-NMR spectrum of **3** in DMSO-d_6.

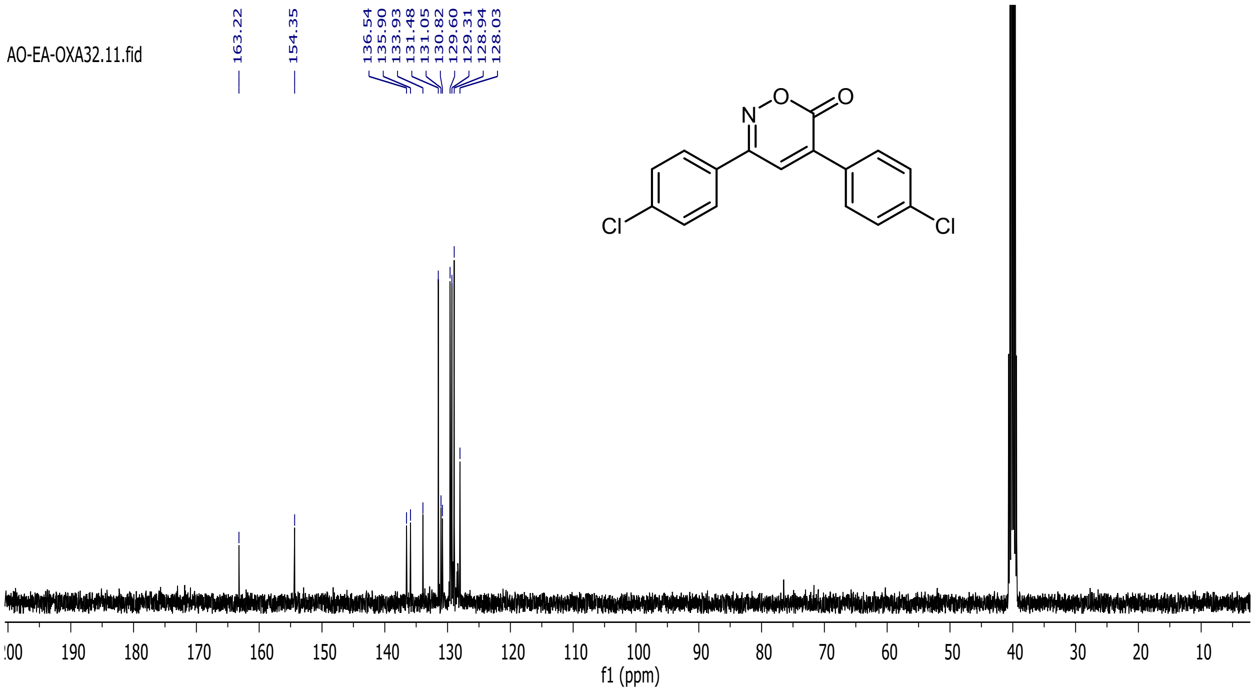

Figure S22. ^{13}C-NMR spectrum of **3** in DMSO-d_6.

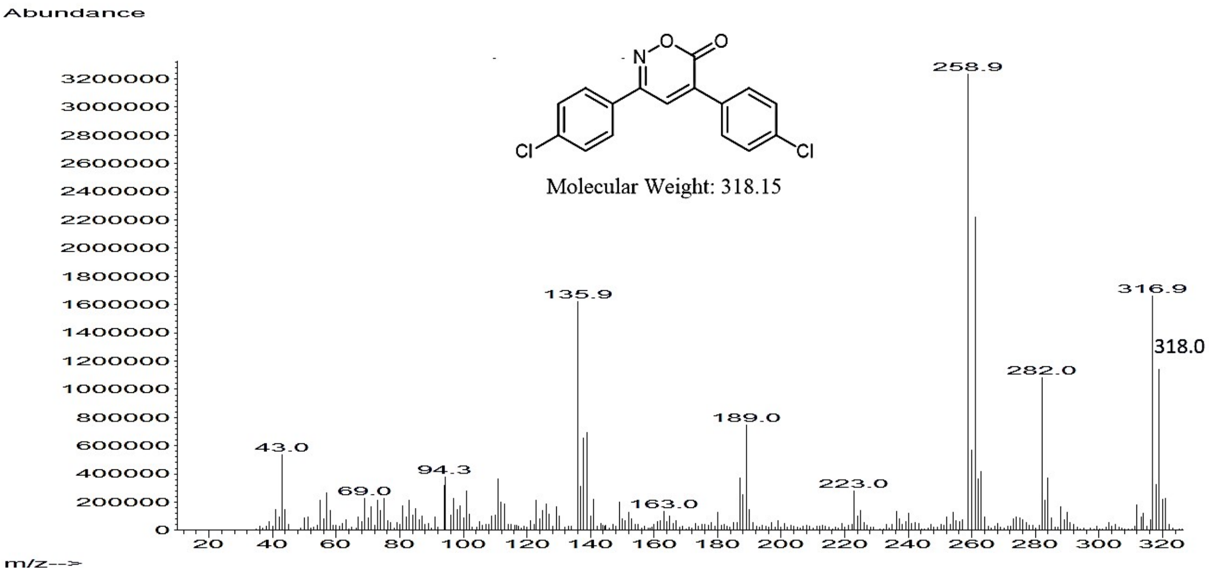

Figure S23. MS(IE) of **3**.

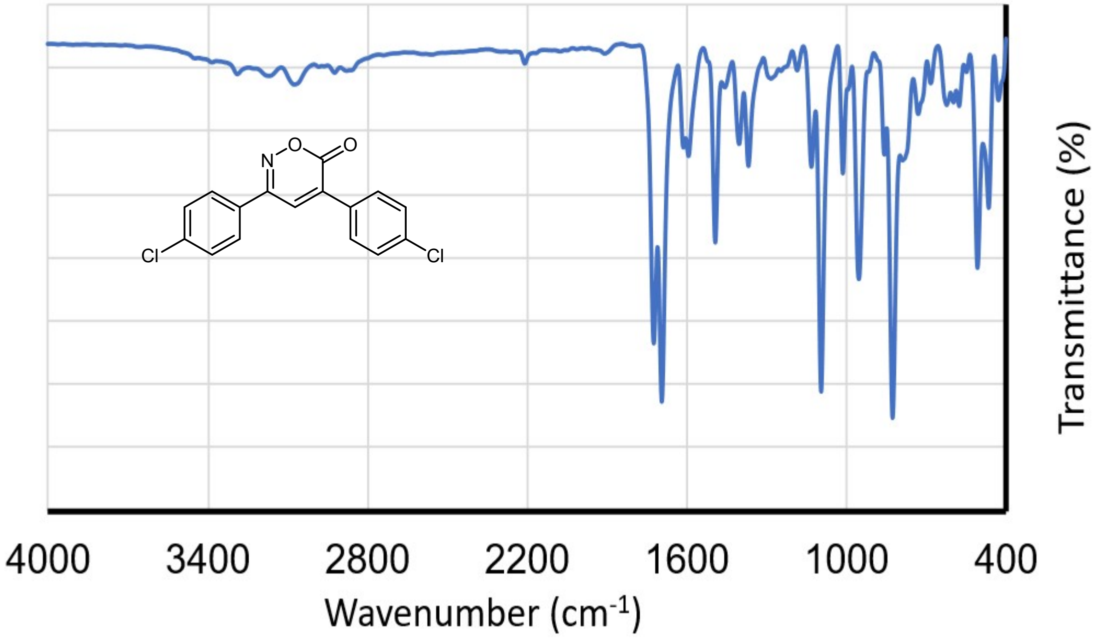

Figure S24. FT-IR of **3**.

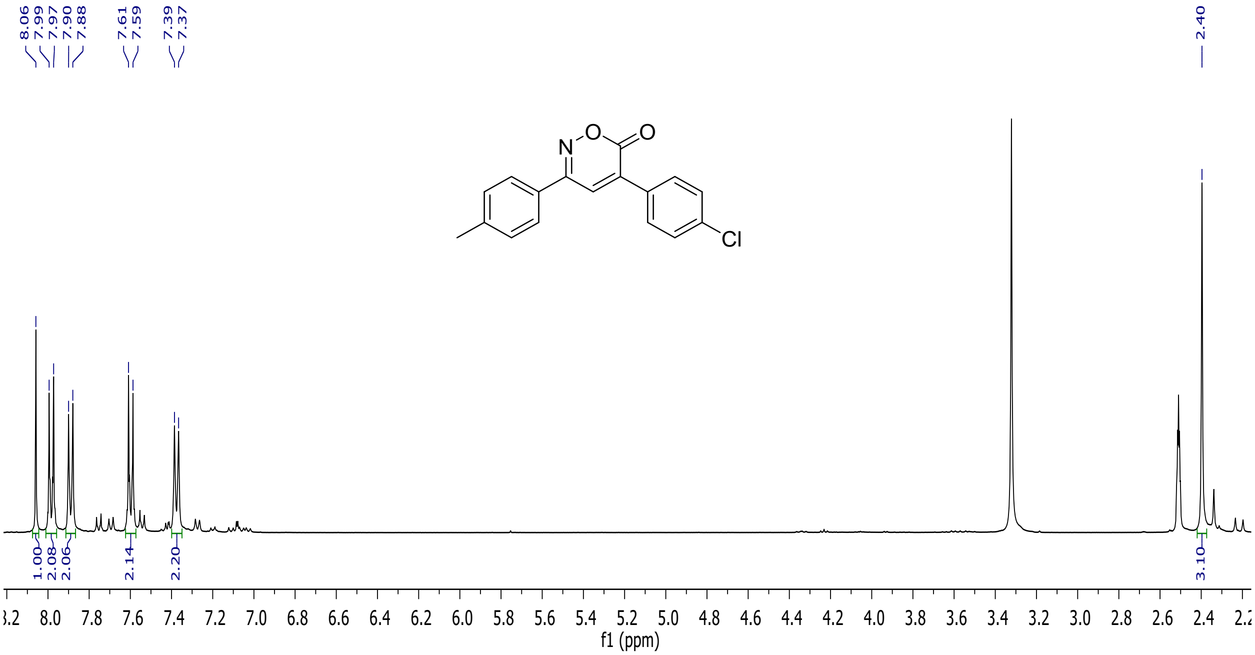

Figure S25. ^{1}H-NMR spectrum of **4** in DMSO-d_6.

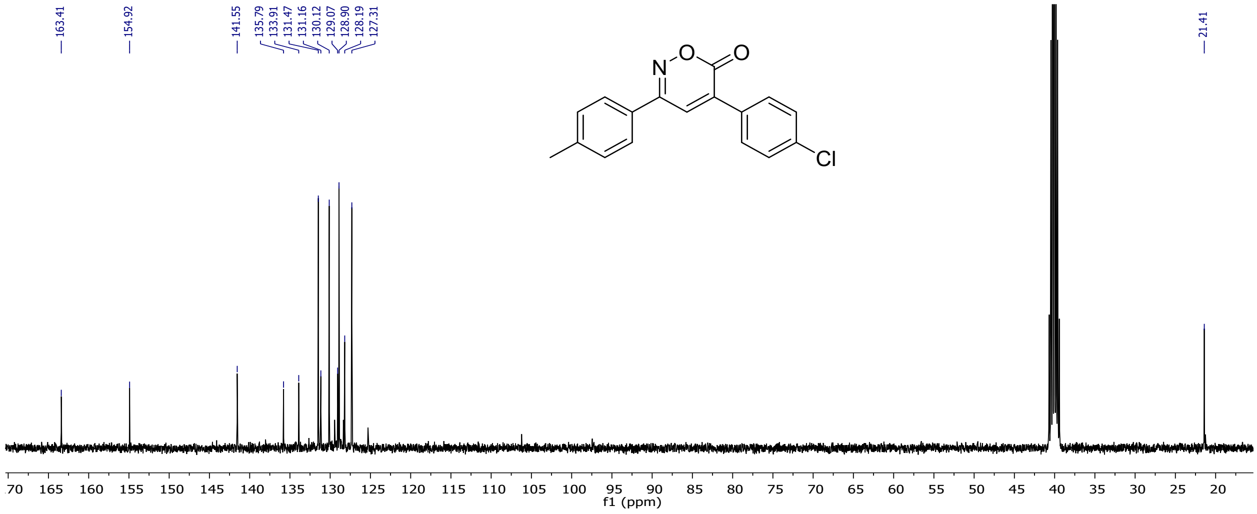

Figure S26. ^{13}C-NMR spectrum of **4** in DMSO-d_6.

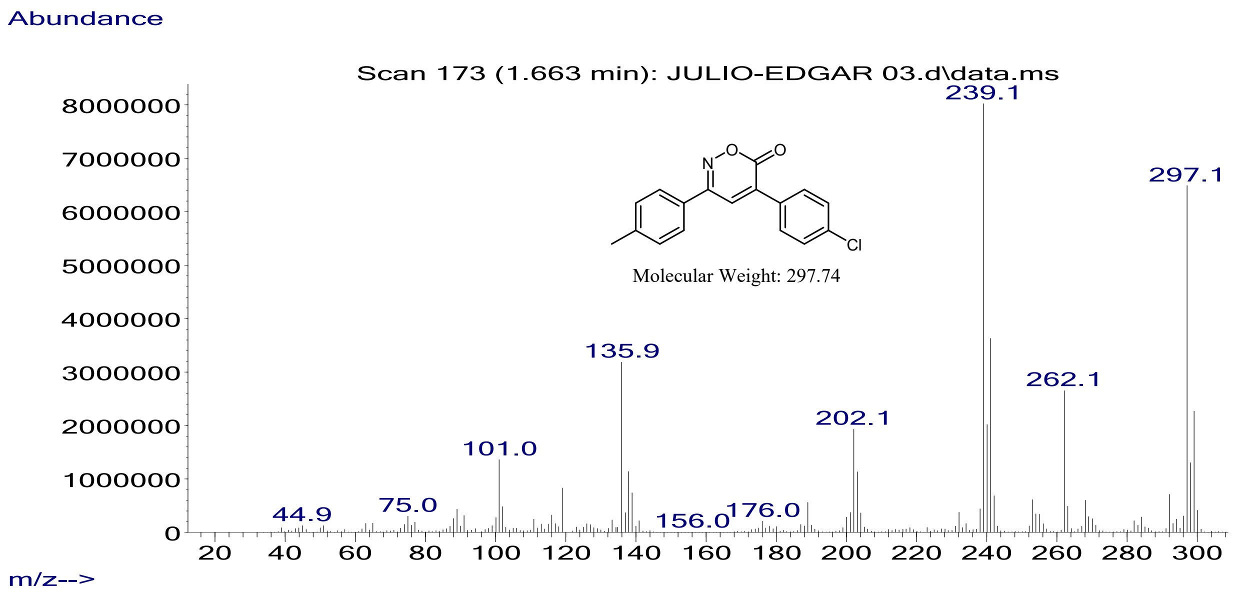

Figure S27. MS(IE) of **4**.

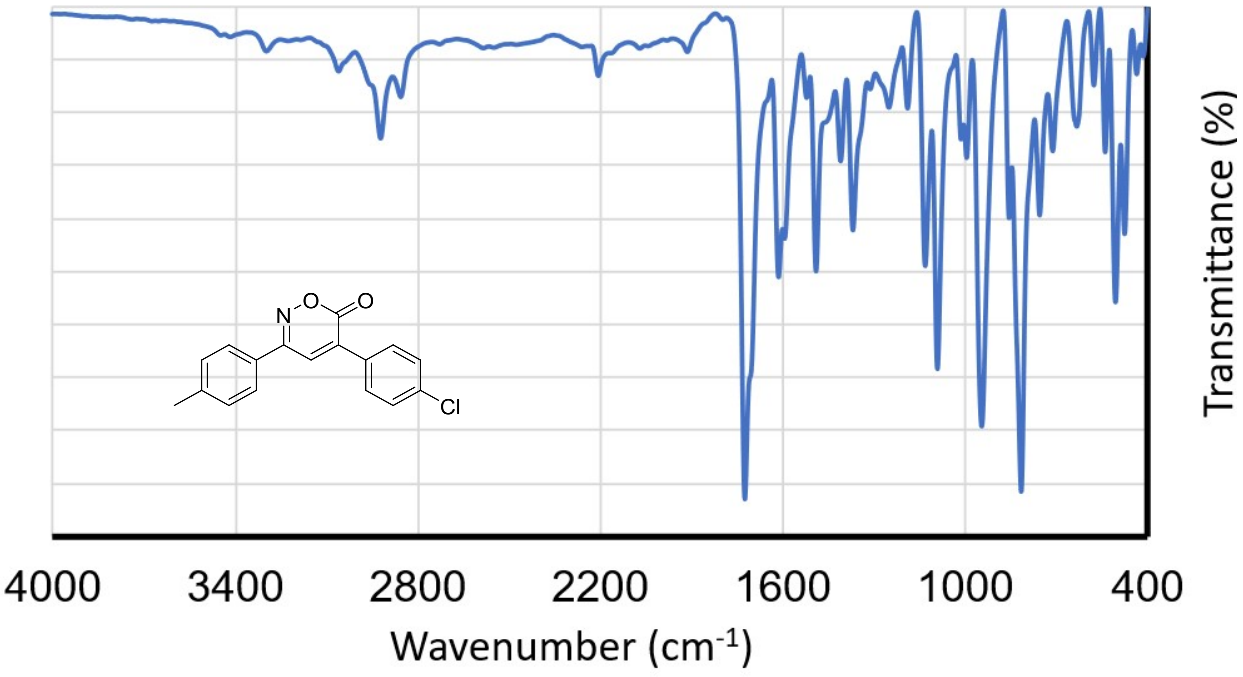

Figure S28. FT-IR of **4**.

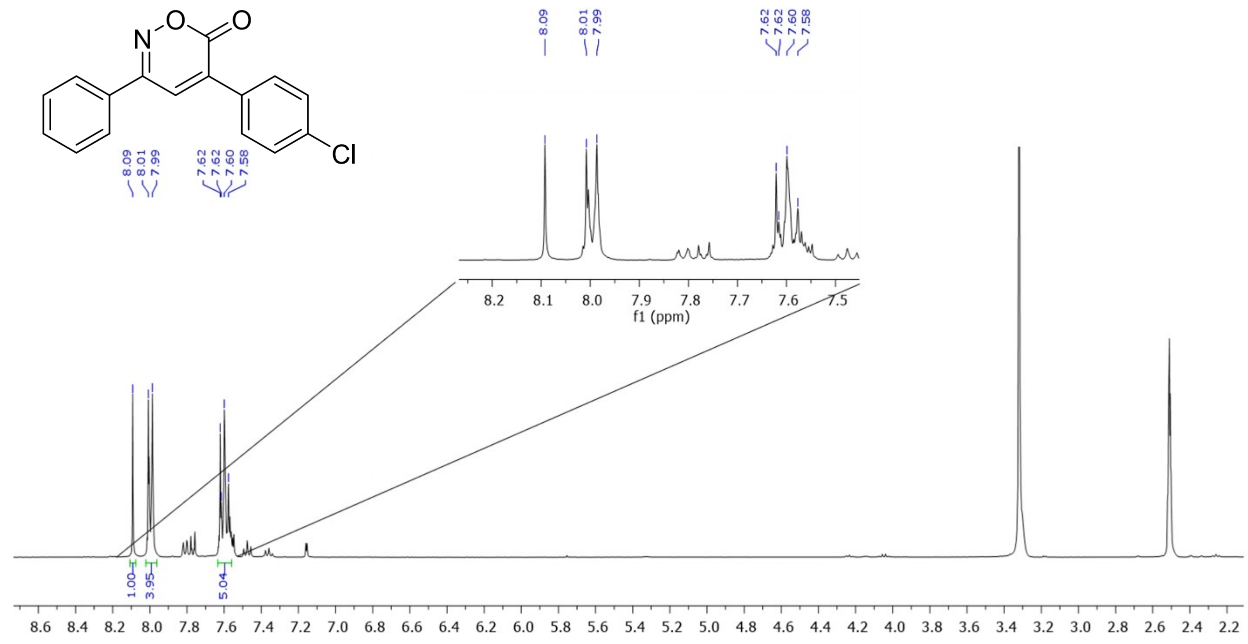

Figure S29. ^{1}H-NMR spectrum of **5** in DMSO-d_6.

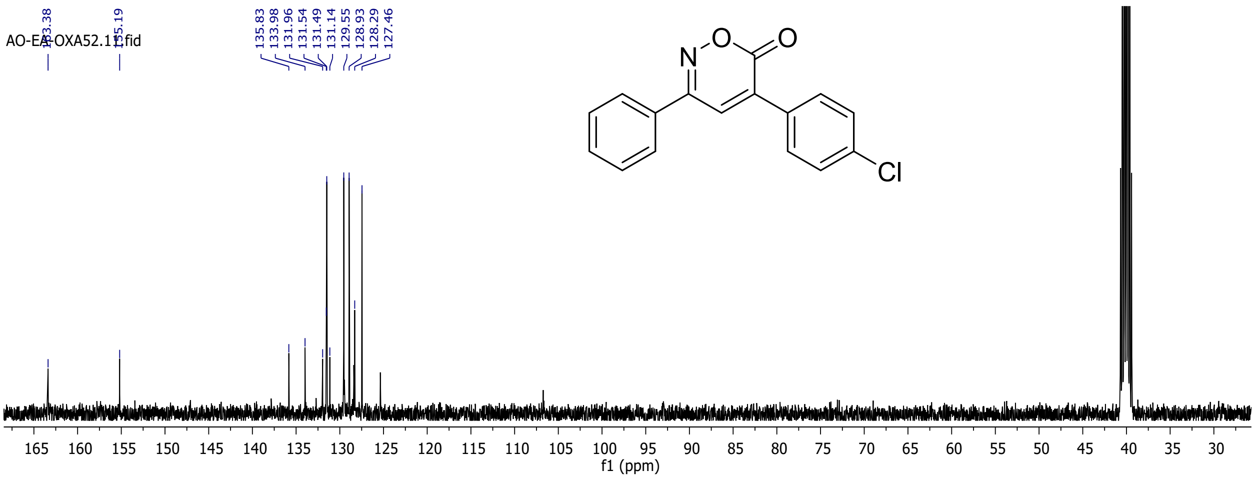

Figure S30. ^{13}C-NMR spectrum of **5** in DMSO-d_6.

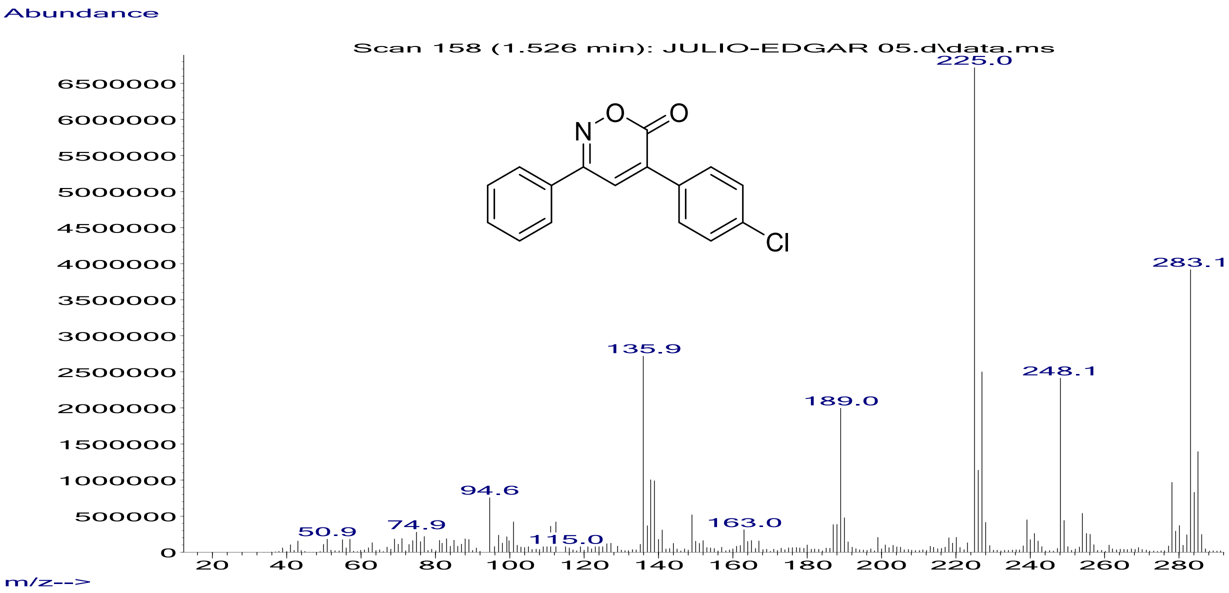

Figure S31. MS(IE) of **5**.

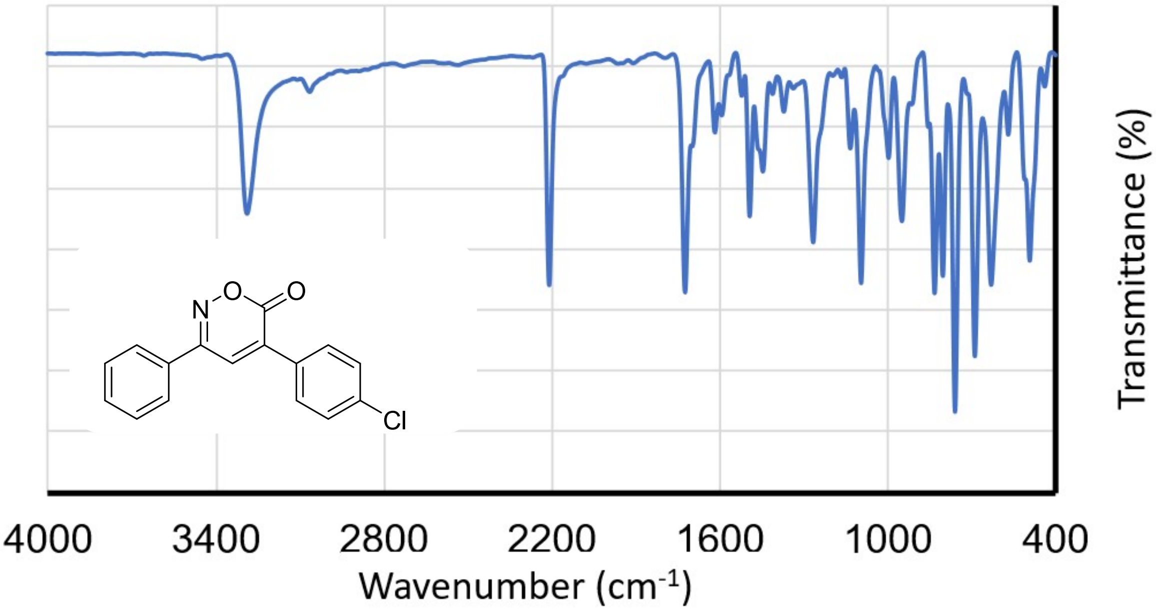

Figure S32. FT-IR of **5**.

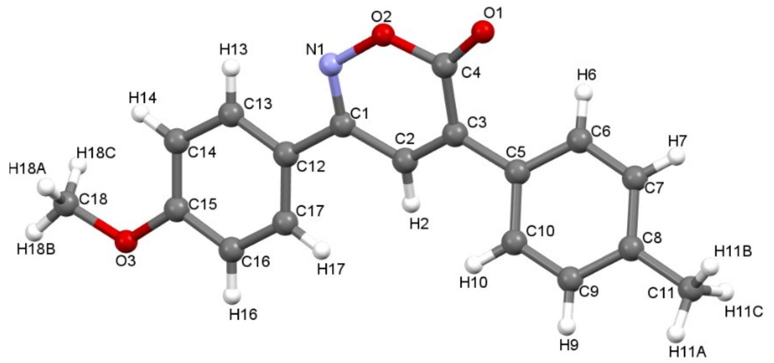

Table S4. Crystal data and structure refinement for OXA-7

Identification code	OXA-7_
Empirical formula	$C_{18}H_{15}NO_3$
Formula weight	293.31
Temperature/K	293(2)
Crystal system	monoclinic
Space group	$P2_1/n$
a/Å	5.0224(7)
b/Å	13.540(2)
c/Å	21.561(3)
α/°	90
β/°	90
γ/°	90
Volume/Å^3	1466.2(4)
Z	4
ρ_{calc}mg/mm^3	1.329
μ/mm^{-1}	0.091
F(000)	616.0
Crystal size/mm^3	0.324 × 0.154 × 0.112
2Θ range for data collection	7.108 to 58.978°
Index ranges	-6 ≤ h ≤ 5, -13 ≤ k ≤ 18, -26 ≤ l ≤ 29
Reflections collected	9792
Independent reflections	3460[R(int) = 0.0403]
Data/restraints/parameters	3460/0/201
Goodness-of-fit on F^2	1.051
Final R indexes [I>=2σ (I)]	R_1 = 0.0566, wR_2 = 0.1209
Final R indexes [all data]	R_1 = 0.1102, wR_2 = 0.1467
Largest diff. peak/hole / e Å^{-3}	0.16/-0.23

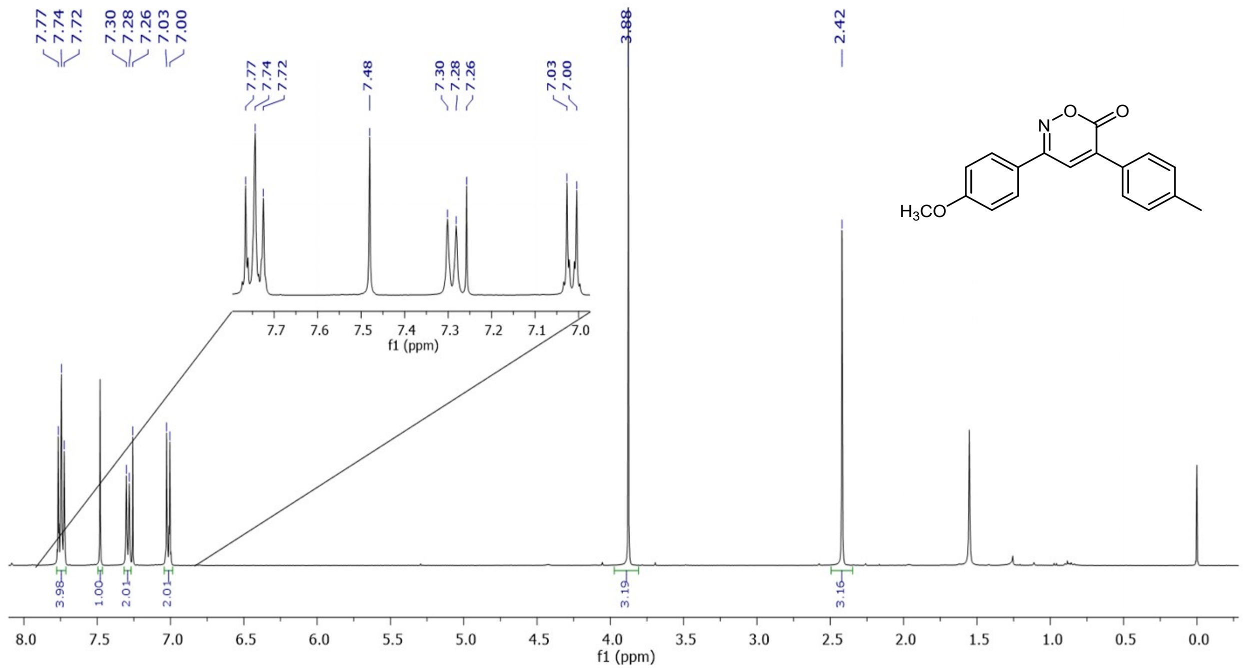

Figure S33. ^{1}H-NMR spectrum of **7** in $CDCl_3$.

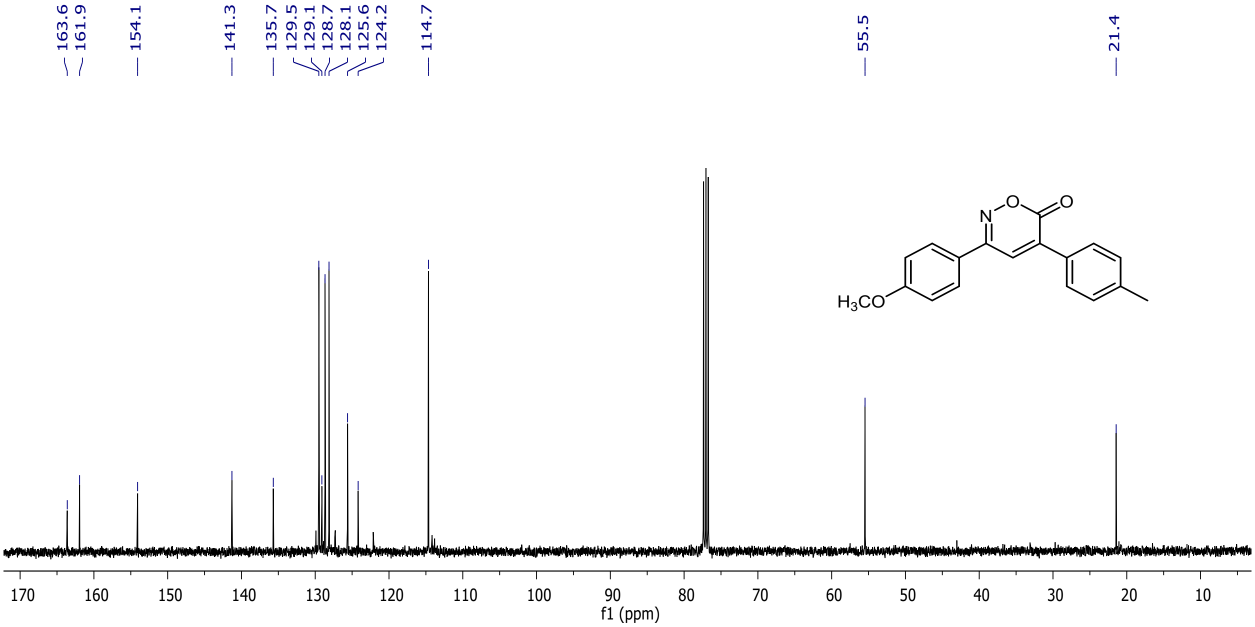

Figure S34. ^{13}C-NMR spectrum of **7** in $CDCl_3$.

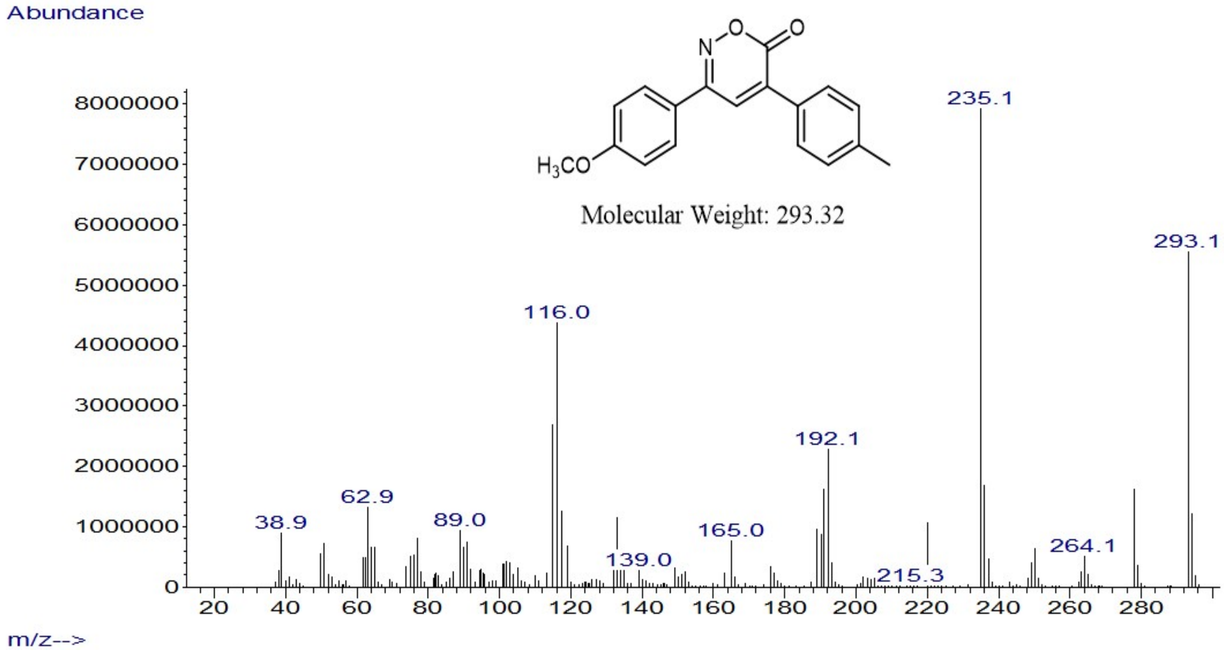

Figure S35. MS(IE) of **7**.

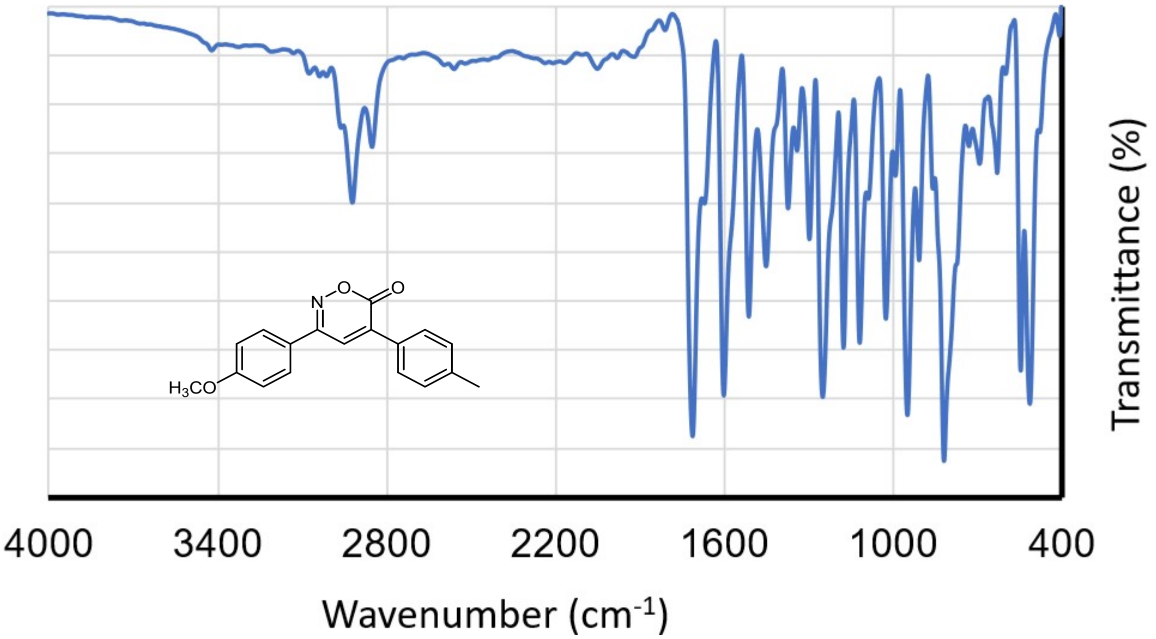

Figure S36. FT-IR of **7**.

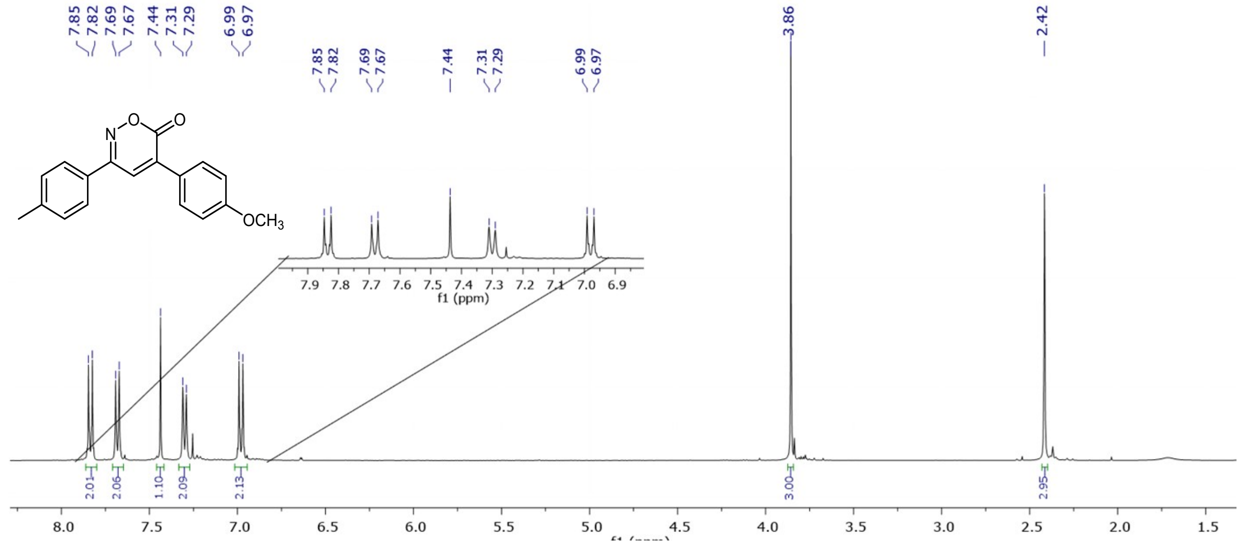

Figure S37. ^{1}H-NMR spectrum of **8** in $CDCl_3$.

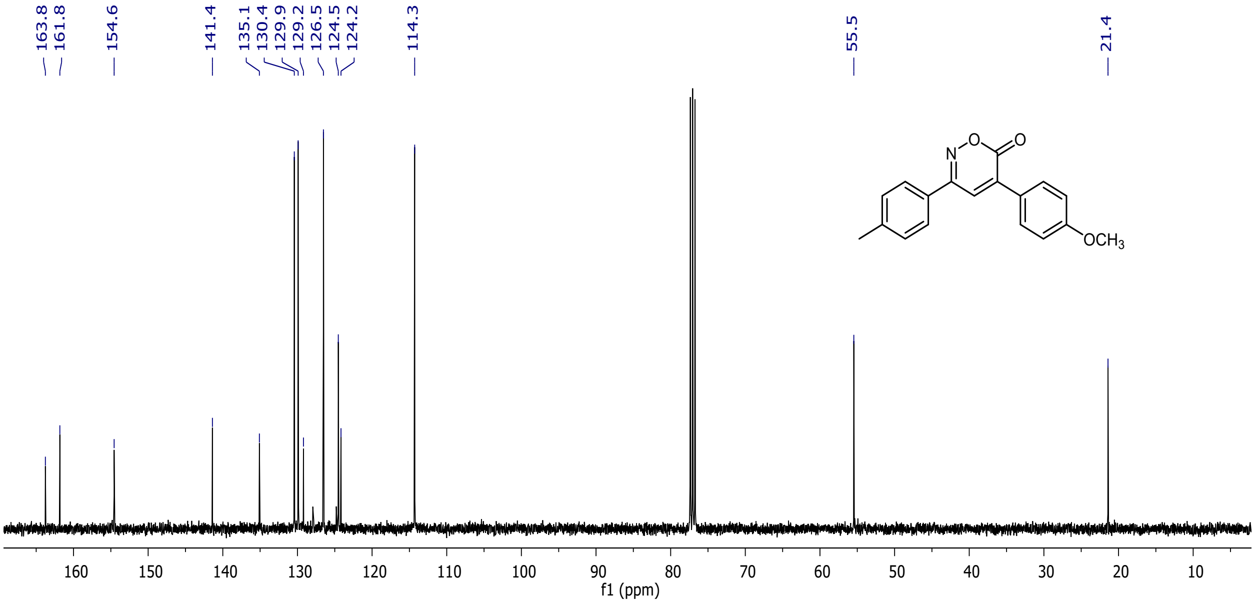

Figure S38. ^{13}C-NMR spectrum of **8** in $CDCl_3$.

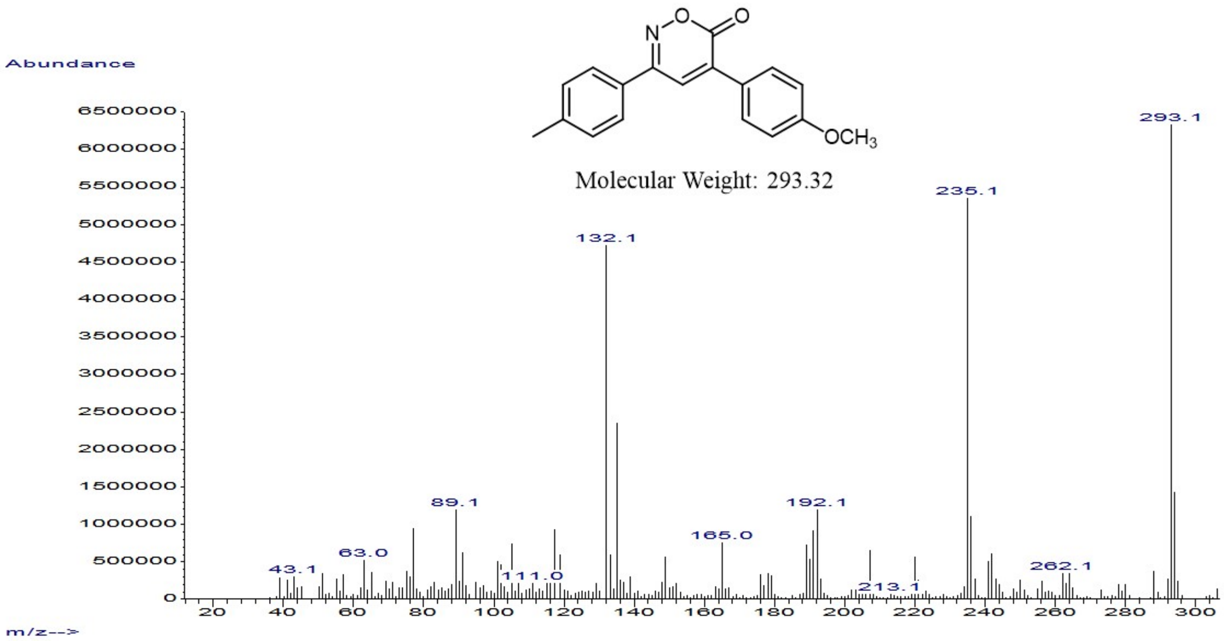

Figure S39. MS(IE) of **8**.

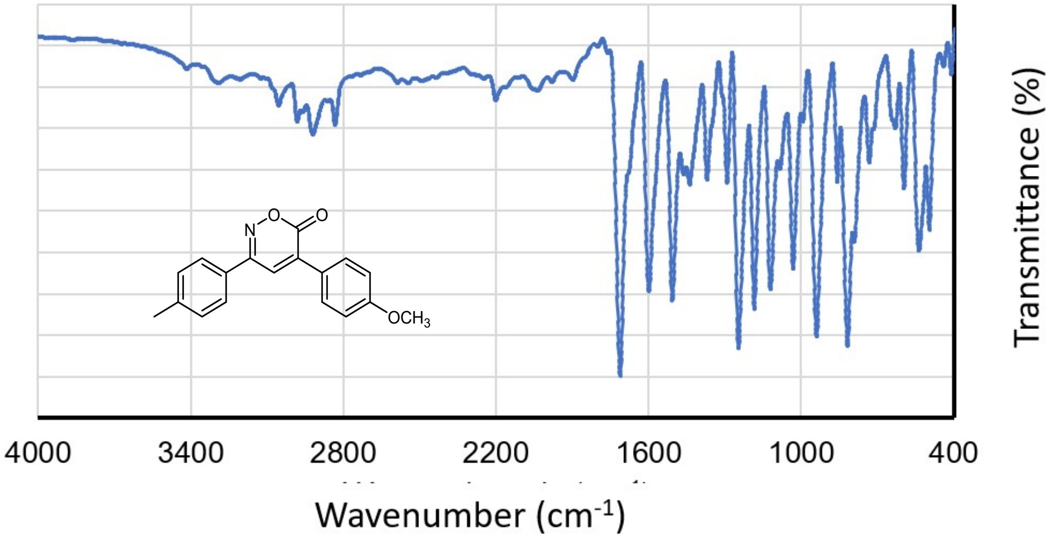

Figure S40. FT-IR of **8**.

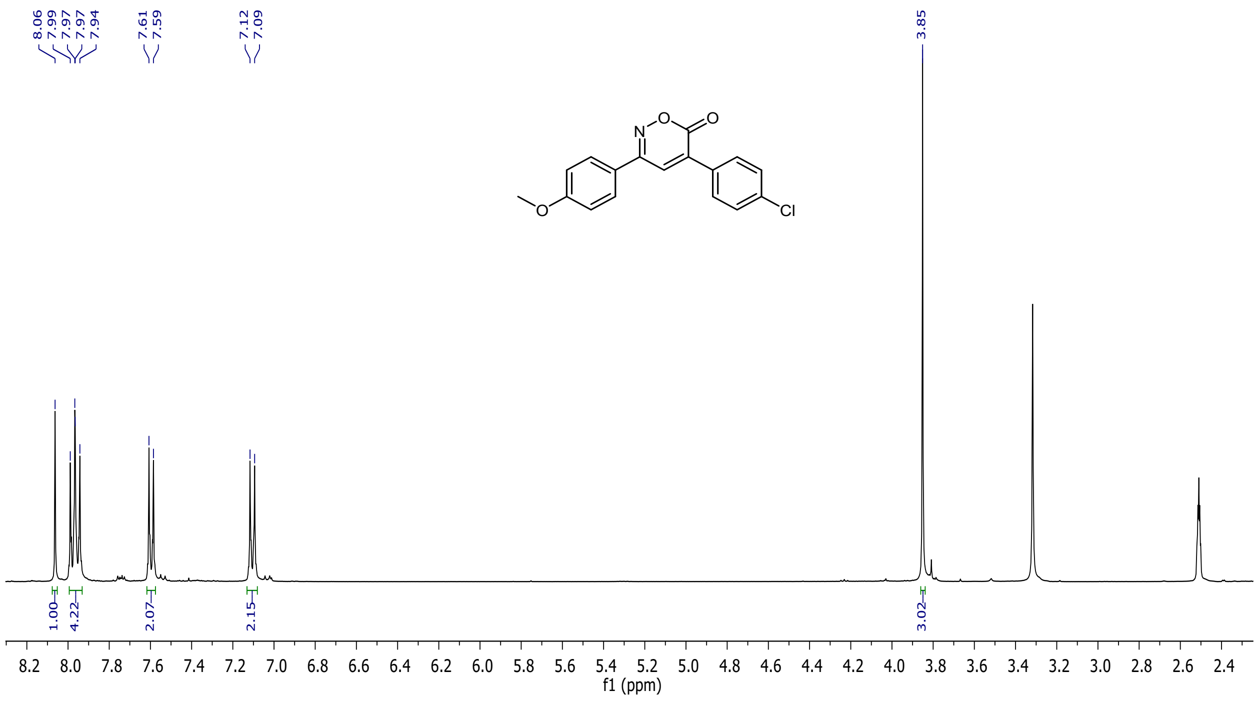

Figure S41. ^{1}H-NMR spectrum of **9** in DMSO-d_6.

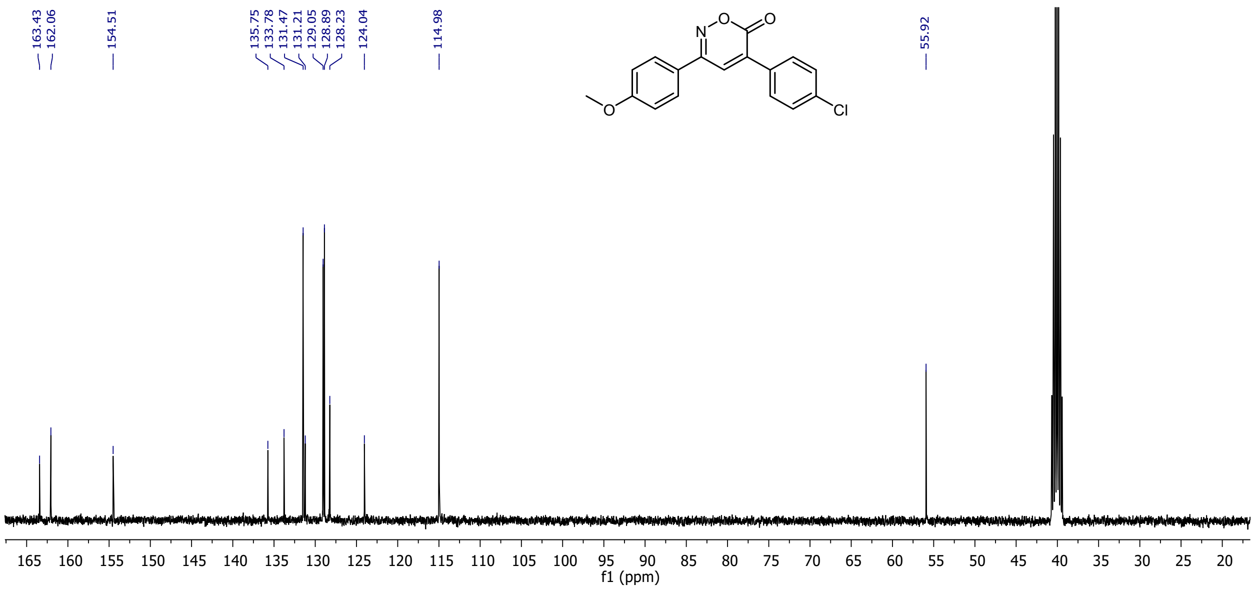

Figure S42. ^{13}C-NMR spectrum of **9** in DMSO-d_6.

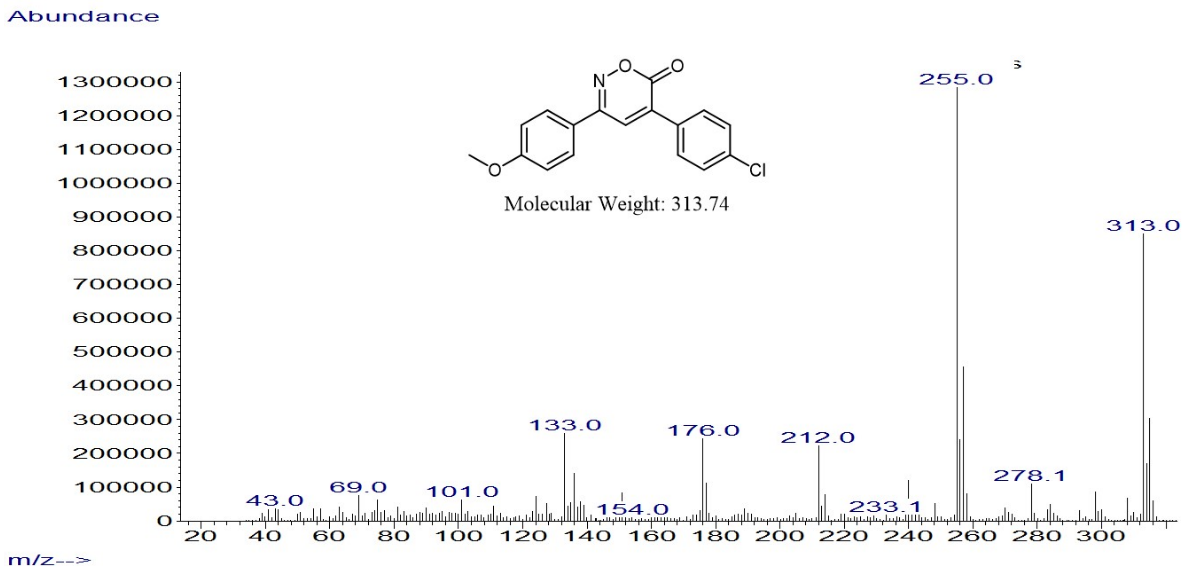

Figure S43. MS(IE) of **9**.

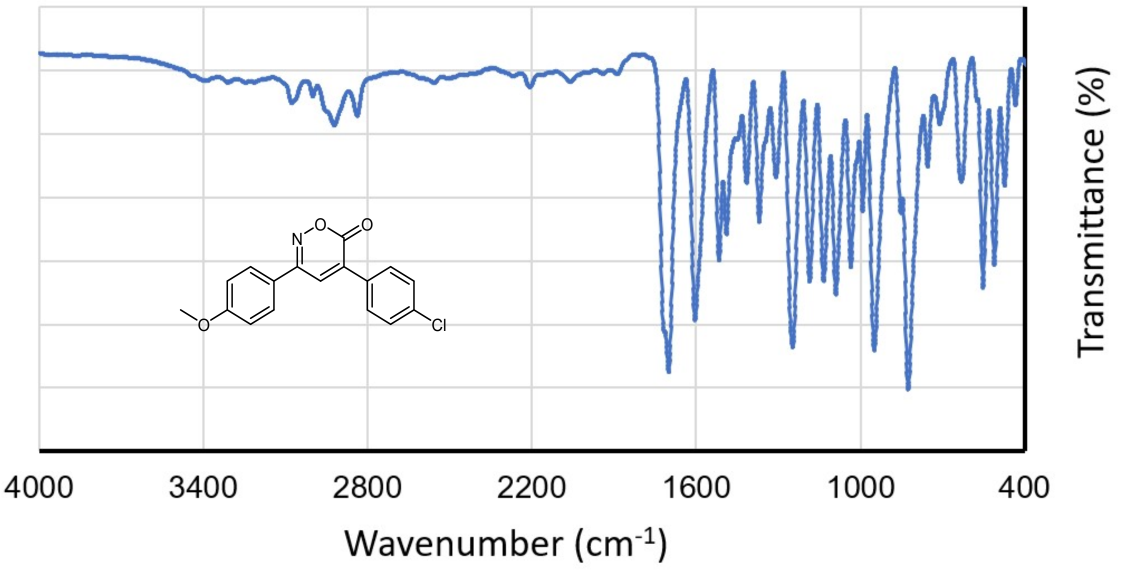

Figure S44. FT-IR of **9**.

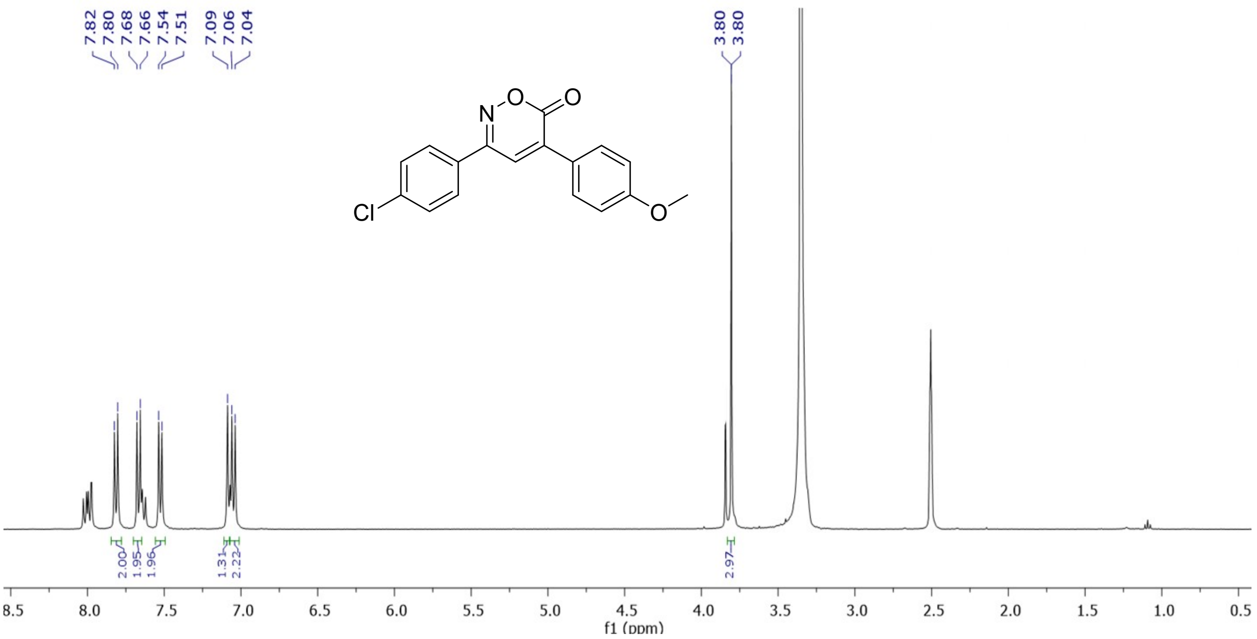

Figure S45. ^{1}H-NMR spectrum of **10** in DMSO-d_6.

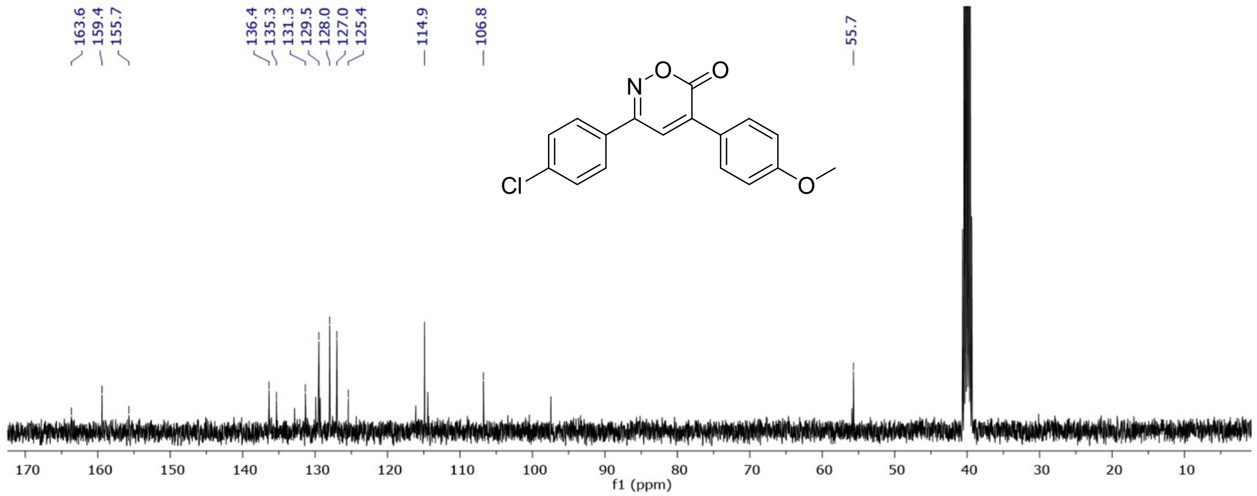

Figure S46. ^{13}C-NMR spectrum of **10** in DMSO-d_6.

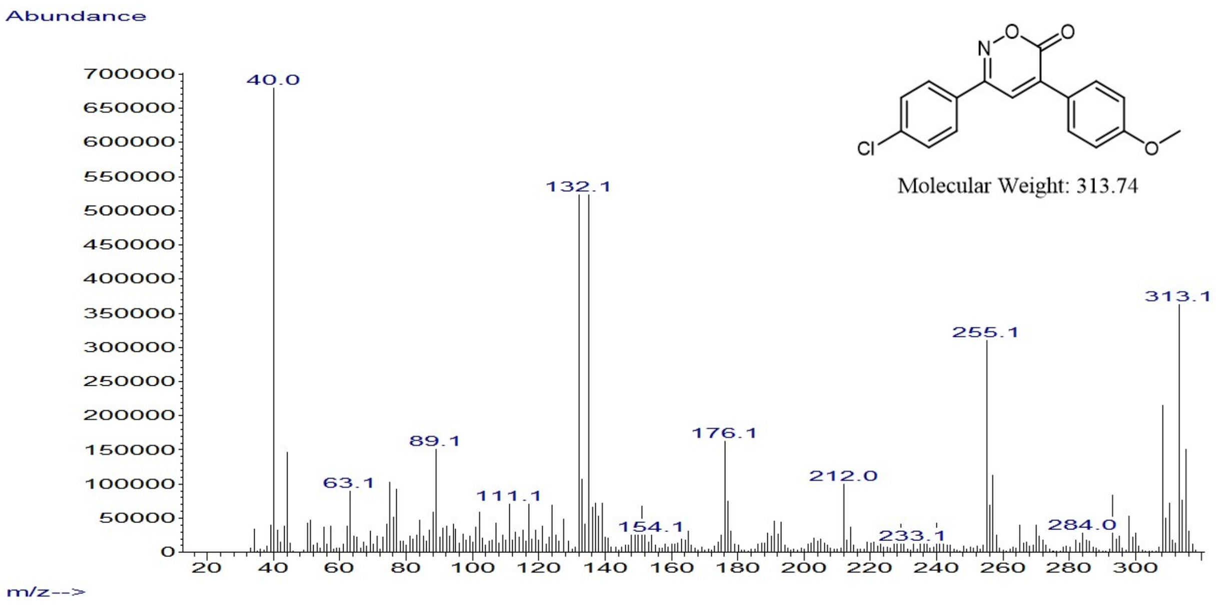

Figure S47. MS(IE) of **10**.

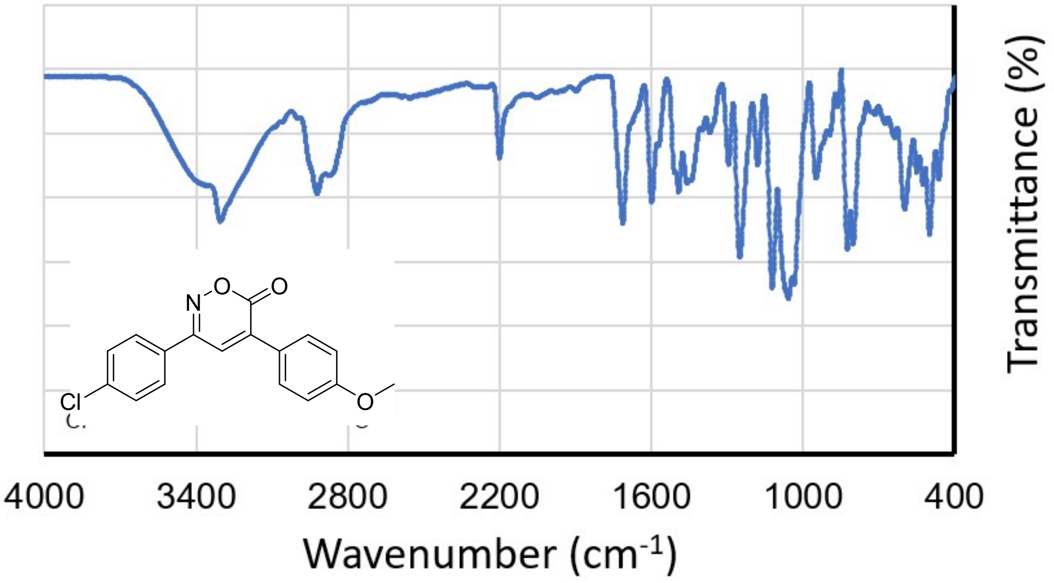

Figure S48. FT-IR of **10**.

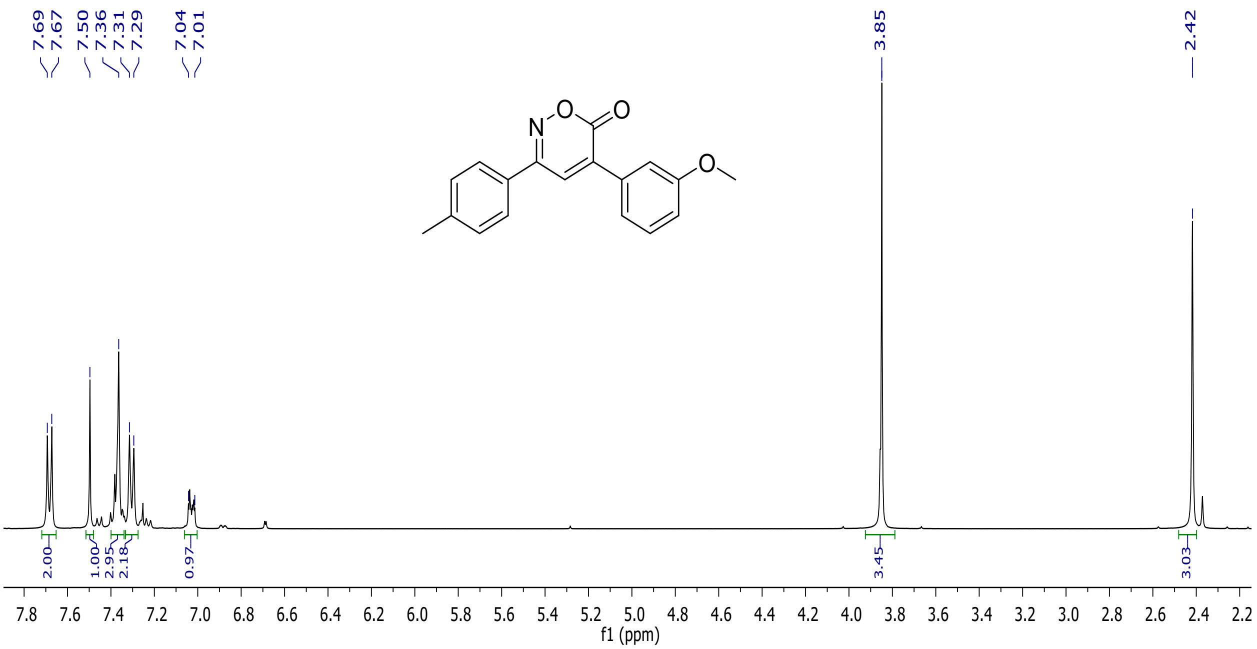

Figure S49. ^{1}H-NMR spectrum of **11** in $CDCl_3$.

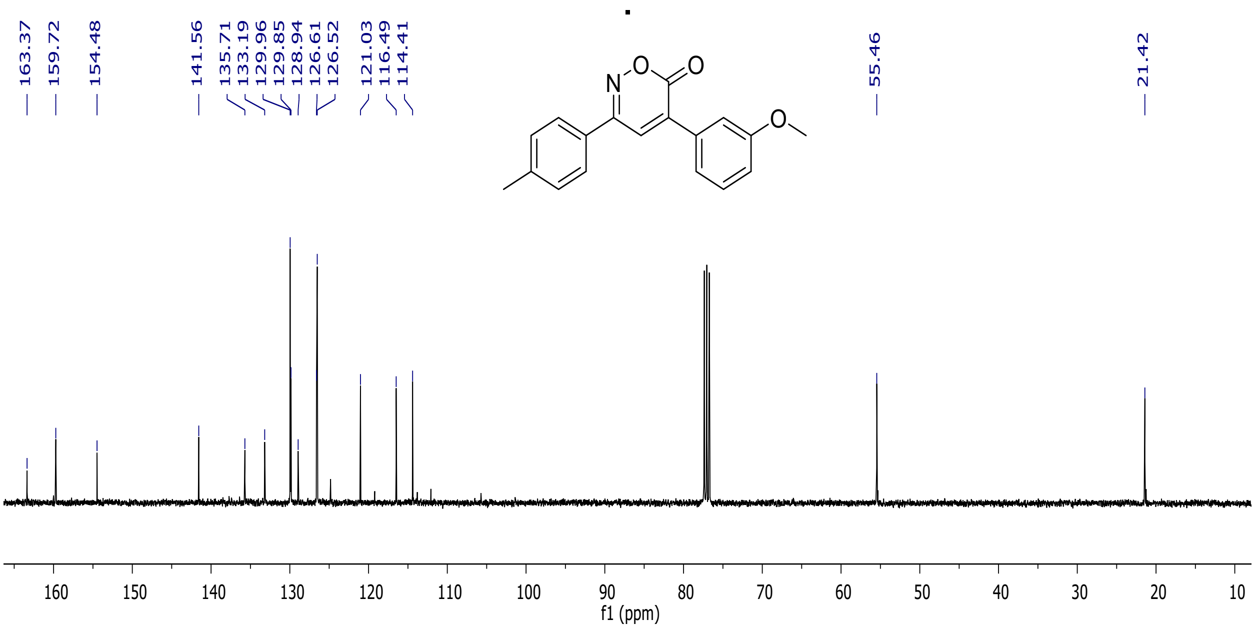

Figure S50. ^{13}C-NMR spectrum of **11** in $CDCl_3$

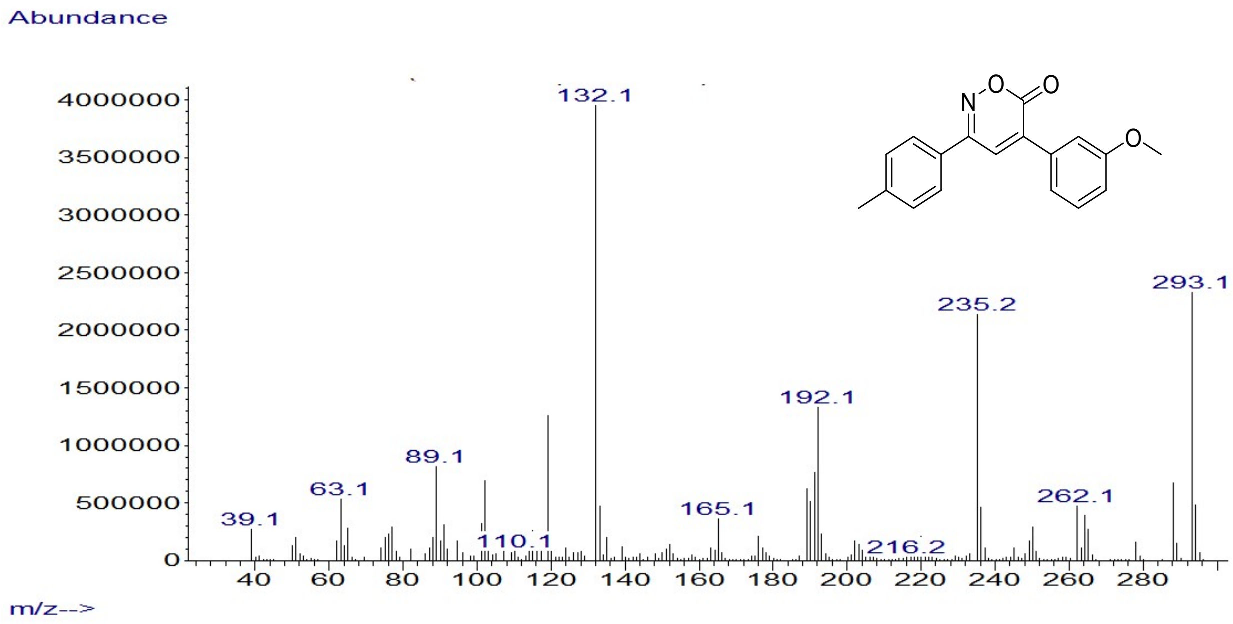

Figure S51. MS(IE) of **11.**

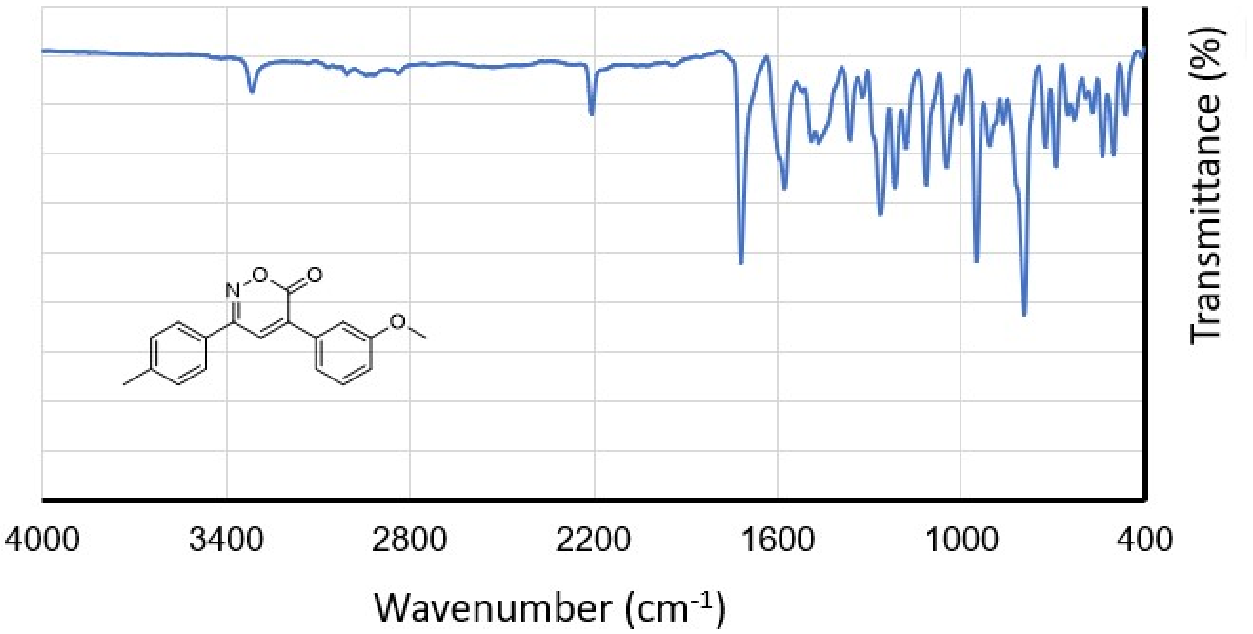

Figure S52. FT-IR of **11**.

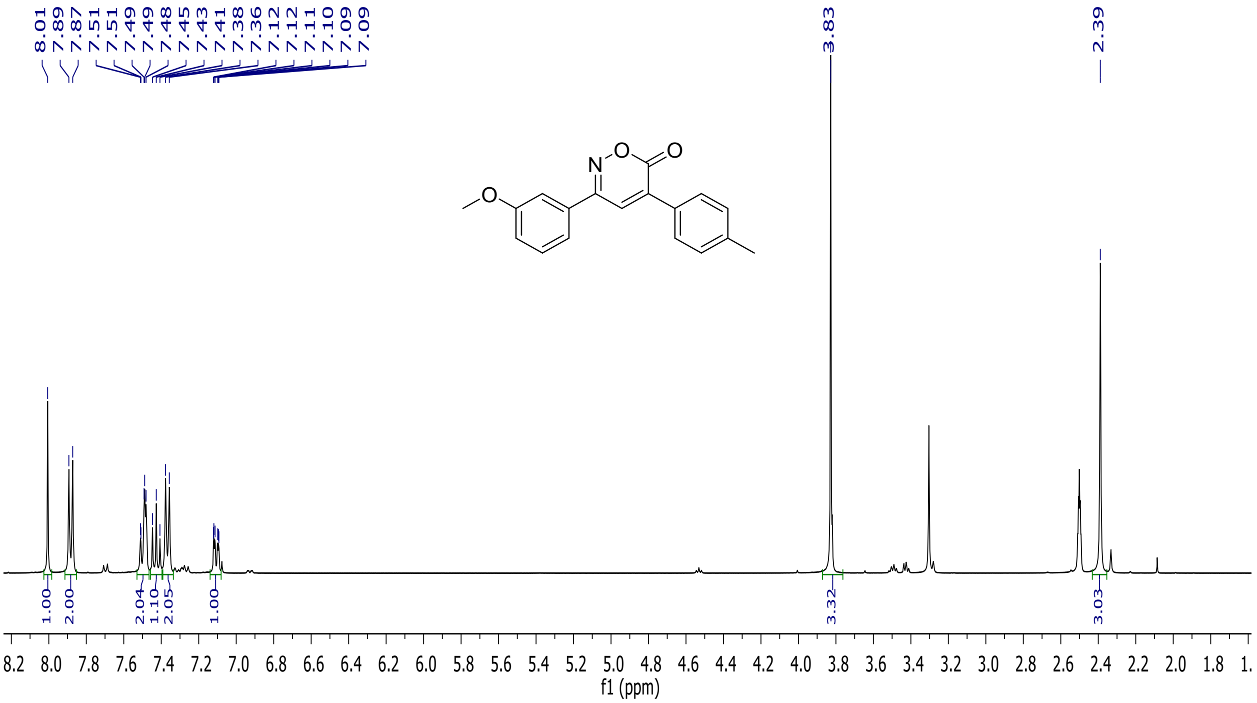

Figure S53. ^{1}H-NMR spectrum of **12** in DMSO-d_6.

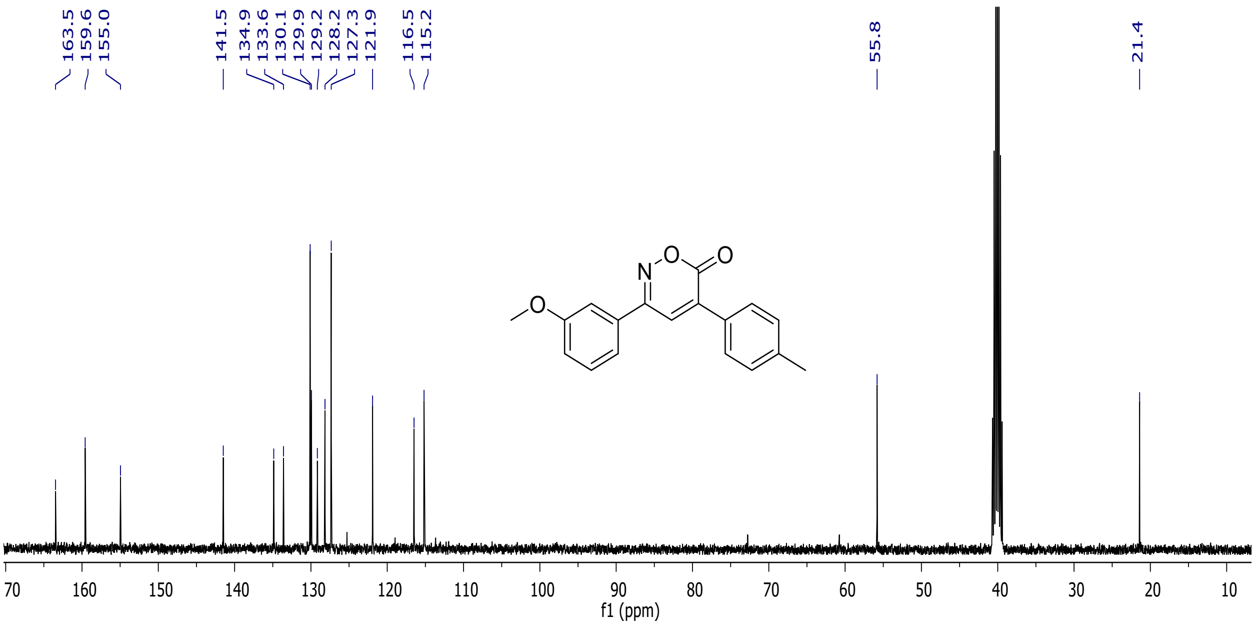

Figure S54. ^{13}C-NMR spectrum of **12** in DMSO-d_6.

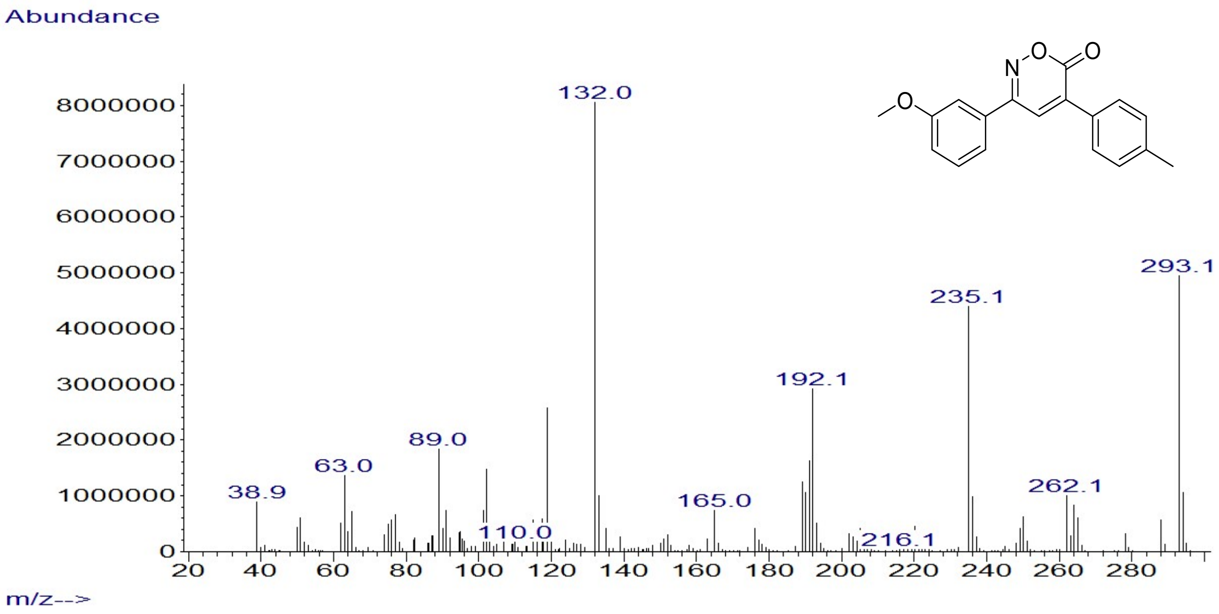

Figure S55. MS(IE) of **12**.

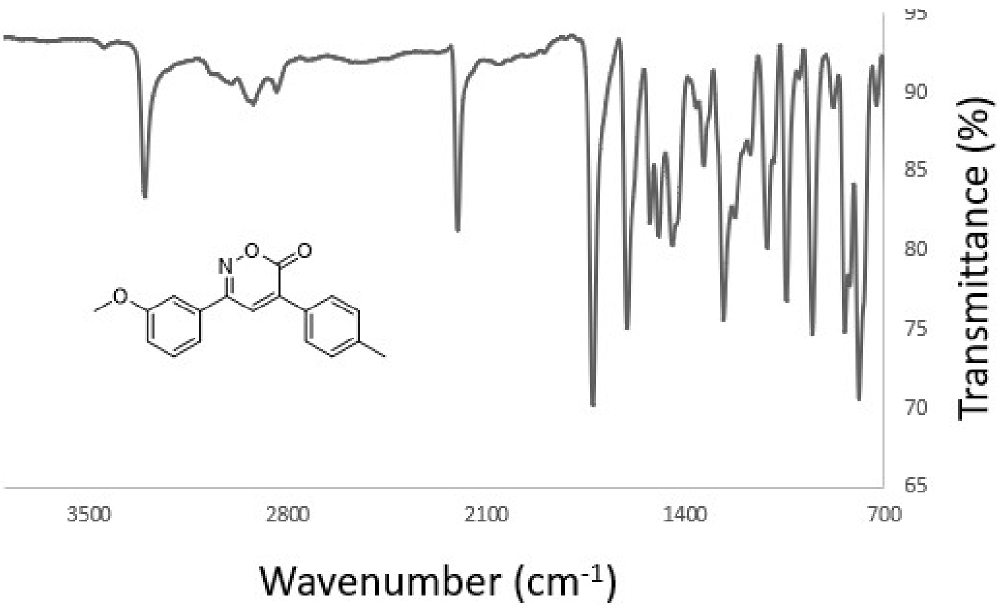

Figure S56. FT-IR of **12**.

Table S5. Bacterial strains used in the antimicrobial test of 6*H*-1,2-oxazin-6-ones.

ATCC strains[a]	Clinically isolated strains[b]
43300 MRSA	MRSA-03
25922	MRSA-04
	MRSA-05

MRSA: Meticillin Resistant *Staphylococcus aureus*. 25922: *Escherichia coli*
a: ATCC strains were obtained from DIFCO Laboratories, Michigan, U.S.A.
b: Clinically isolated strains were donated by Laboratorio de Bacteriología del Instituto Nacional de Pediatría de México, Ciudad de México, México.

Table S6. MIC (µg/mL) of Linezolid and Gentamicin against bacterial strains used in the study.

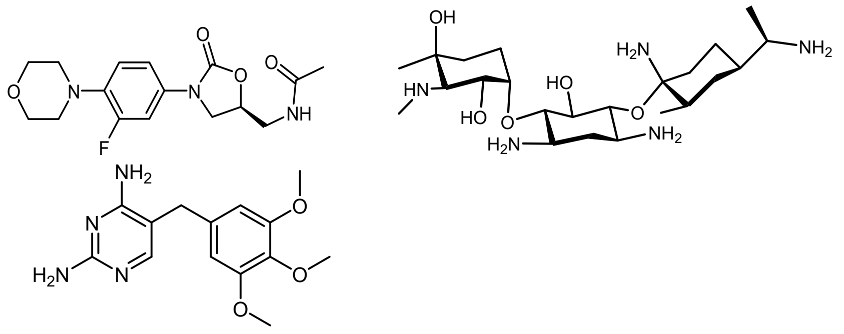

Bacterial	Linezolid	Gentamicin	Trimethoprim
43300 MRSA	4	2	1
25922	>50	1	>50
MRSA-03	4	2	1
MRSA-04	8	2	2
MRSA-05	8	1	2

Table S7. Determination *in vitro* toxicity of 6*H*-1,2-oxazin-6-ones by the *Artemia salina* model.

Compounds	DLT	ALT	% Mortality	% Vitality
**1**	0	10	0	100
**2**	0	10	0	100
**3**	0	10	0	100
**4**	0	10	0	100
**6**	0	10	0	100
**8**	0	10	0	100

% Mortality = DLT/ ALT x 100 %, DLT is the number of dead Larvae in the tube, ALT is the number of alive larvae in the tube
